# Does greater individual social capital improve the management of hypertension? Cross-national analysis of 61 229 individuals in 21 countries

**DOI:** 10.1136/bmjgh-2017-000443

**Published:** 2017-12-17

**Authors:** Benjamin Palafox, Yevgeniy Goryakin, David Stuckler, Marc Suhrcke, Dina Balabanova, Khalid F Alhabib, Alvaro Avezum, Ahmad Bahonar, Xiulin Bai, Jephat Chifamba, Antonio L Dans, Rafael Diaz, Rajeev Gupta, Romaina Iqbal, Noorhassim Ismail, Manmeet Kaur, Mirac V Keskinler, Rasha Khatib, Annamarie Kruger, Iolanthe M Kruger, Fernando Lanas, Scott A Lear, Wei Li, Jia Liu, Patricio Lopez-Jaramillo, Nasheeta Peer, Paul Poirier, Omar Rahman, Rajamohanan K Pillai, Sumathy Rangarajan, Annika Rosengren, Sumathi Swaminathan, Andrzej Szuba, Koon Teo, Yang Wang, Andreas Wielgosz, Karen E Yeates, Afzalhussein Yusufali, Salim Yusuf, Martin McKee

**Affiliations:** 1 The Centre for Global Chronic Conditions, London School of Hygiene and Tropical Medicine, London, UK; 2 Organization for Economic Cooperation and Development, Paris, France; 3 Department of Policy Analysis and Public Management and Dondena Research Centre, University of Bocconi, Milan, Italy; 4 Centre for Health Economics, University of York, York, UK; 5 Department of Cardiac Sciences, King Fahad Cardiac Center, College of Medicine, King Saud University, Riyadh, Saudi Arabia; 6 Dante Pazzanese Institute of Cardiology, São Paulo, Brazil; 7 Cardiovascular Research Institute, Isfahan University of Medical Sciences, Isfahan, The Islamic Republic of Iran; 8 National Center for Cardiovascular Disease, Fuwai Hospital, Peking Union Medical College & Chinese Academy of Medical Sciences, Beijing, China; 9 College of Health Sciences, University of Zimbabwe, Harare, Zimbabwe; 10 UP College of Medicine, University of the Philippines Manila, Manila, Philippines; 11 Estudios Clinicos Latino America, Rosario, Argentina; 12 Eternal Heart Care Centre and Research Institute, Jaipur, India; 13 Departments of Community Health Sciences and Medicine, Aga Khan University, Karachi, Pakistan; 14 Department of Community Health, University Kebangsaan Malaysia, Kuala Lumpur, Malaysia; 15 School of Public Health, Postgraduate Institute of Medical Education and Research, Chandigarh, India; 16 Department of Internal Medicine, Istanbul Medeniyet University Goztepe Training and Research Hospital, Istanbul, Turkey; 17 Department of Public Health Sciences, Loyola University Medical Center, Maywood, Illinois, USA; 18 Africa Unit for Transdisciplinary Health Research, North-West University, Potchefstroom, South Africa; 19 Universidad de La Frontera, Temuco, Chile; 20 Simon Fraser University, Burnaby, British Columbia, Canada; 21 Research Institute FOSCAL, Santander, Colombia; 22 South African Medical Research Council, Durban, South Africa; 23 Institut universitaire de cardiologie et de pneumologie de Quebec, Quebec, Canada; 24 Independent University, Dhaka, Bangladesh; 25 Department of Pediatrics, Dr SMCSI Medical College Karakonam, Trivandrum, India; 26 Population Health Research Institute, Hamilton Health Sciences, Hamilton, Ontario, Canada; 27 Department of Molecular and Clinical Medicine, University of Gothenburg, Goteborg, Sweden; 28 St John’s Research Institute, Bangalore, Karnataka, India; 29 Division of Angiology, Wroclaw Medical University, Wroclaw, Poland; 30 University of Ottawa, Ottawa, Ontario, Canada; 31 Department of Medicine, Queen’s University, Kingston, Ontario, Canada; 32 Hatta Hospital, Dubai Health Authority/Dubai Medical University, Dubai, United Arab Emirates

**Keywords:** hypertension, health economics, epidemiology, control strategies, health systems

## Abstract

**Introduction:**

Social capital, characterised by trust, reciprocity and cooperation, is positively associated with a number of health outcomes. We test the hypothesis that among hypertensive individuals, those with greater social capital are more likely to have their hypertension detected, treated and controlled.

**Methods:**

Cross-sectional data from 21 countries in the Prospective Urban and Rural Epidemiology study were collected covering 61 229 hypertensive individuals aged 35–70 years, their households and the 656 communities in which they live. Outcomes include whether hypertensive participants have their condition detected, treated and/or controlled. Multivariate statistical models adjusting for community fixed effects were used to assess the associations of three social capital measures: (1) membership of any social organisation, (2) trust in other people and (3) trust in organisations, stratified into high-income and low-income country samples.

**Results:**

In low-income countries, membership of any social organisation was associated with a 3% greater likelihood of having one’s hypertension detected and controlled, while greater trust in organisations significantly increased the likelihood of detection by 4%. These associations were not observed among participants in high-income countries.

**Conclusion:**

Although the observed associations are modest, some aspects of social capital are associated with better management of hypertension in low-income countries where health systems are often weak. Given that hypertension affects millions in these countries, even modest gains at all points along the treatment pathway could improve management for many, and translate into the prevention of thousands of cardiovascular events each year.

**Key questions**

**What is already known about this topic?**

- Only two previous studies have examined social capital’s effect on the management of hypertension, both of which combined multiple measures of social capital to derive an overall index of an individual’s social capital for use in their analyses.
- A Finnish study of hypertensive employees found no association between social capital and adherence to antihypertensive medication; and another cross-sectional study of hypertensive adults in India, Pakistan and Bangladesh produced no evidence for an association between social capital and any of the three key stages of hypertension management: the likelihood of diagnosis, use of antihypertensive medication or control.

**What are the new findings?**

- Our study examines the same three outcomes as the second study reported above; but contrary to previous studies, we show that social capital is associated with the management of hypertension.
- Consistent with leading theoretical conceptualisations of social capital that identify distinct forms with different causal mechanisms, only some forms of social capital were found to modestly increase the likelihood of having one’s hypertension detected and controlled, but not treated with medications.
- Crucially, these associations were only present among participants from low-income countries where health systems may be less well-equipped to provide care for chronic conditions.

**Recommendations for policy**

- Given that cardiovascular disease is now the leading cause of death worldwide, with hypertension as the key modifiable risk factor, our findings provide insight to policy makers and planners on how to leverage social capital to benefit public health.
- This is important as improving population-level hypertension control requires a multifaceted approach.
- This evidence is of particular relevance for low-income countries where health budgets are constrained, because interventions based on social capital could be envisaged that would promote inclusiveness and cross-sectoral participation at relatively low cost.

## Introduction

Hypertension is a leading avoidable cause of cardiovascular disease-related deaths worldwide.^1^ Evidence that hypertension control is inadequate in many settings calls for better understanding of how to improve detection, treatment and control, especially in resource-deprived settings.

One factor that could promote better patient management of hypertension is social capital. The World Bank defines social capital as the ’internal social and cultural coherence of society, arising from the norms and values that govern interactions among people and the institutions in which they are embedded'.^2^ It has long been recognised as a contributor to economic growth and overall human well-being, with several studies confirming its beneficial association with health outcomes, including self-reported health, depression and all-cause mortality.^3–7^ Social capital acts at the individual and community levels and is often characterised by three dimensions^8^: (1) *bonding*, referring to the connections within networks of family members and helps individuals access care and resources; (2) *bridging*, which expands social networks through friends of friends and may help individuals get ahead in life and (3) *linking*, which involves connections across social classes and may help individuals access resources that they otherwise would not be able to.^4 6^

In theory, the ability of social capital to help people obtain information and services could help hypertensive patients overcome barriers to attaining care. As few experience symptoms, they must be able to access services where their condition can be detected and regularly monitored.^9^ Both lifestyle changes and medicine adherence can be facilitated by support from friends and family, including potentially the means of accessing and affording medicines.^10^ There are many ways in which the information and support networks that characterise social capital could help hypertensive patients to overcome the many barriers they face,^11^ ranging from lack of knowledge of the consequences of hypertension and its treatment, lack of motivation, social pressures, stress and anxiety and the effects of poor memory.

In this analysis, we test the hypothesis that greater individual social capital improves rates of detection, use of treatment and overall control of hypertension. We focus on the bridging and linking types of social capital that extend beyond the home and family, as there is already a body of research on bonding capital, regarding how families provide care and support for loved ones with chronic disease and this is less amenable to intervention.^5 11^ We further hypothesise that bridging and linking aspects of social capital will be more important in areas where health systems are weak.

## Methods

### Data

The Prospective Urban and Rural Epidemiology (PURE) study is a large global study of cardiovascular disease incidence, mortality and risk factors.^12^ Data are collected from urban and rural communities within 21 countries including 4 high-income countries (HICs) (Canada, Sweden, Saudi Arabia, United Arab Emirates); 7 upper-middle-income countries (UMICs) (Argentina, Brazil, Chile, Poland, Malaysia, South Africa, Turkey); 5 lower-middle-income countries (LMICs) (China, Colombia, Iran, Occupied Palestinian Territory, Philippines) and 5 low-income countries (LICs) (Bangladesh, India, Pakistan, Tanzania, Zimbabwe). The selection of countries in PURE was designed to achieve a balance between different economic levels, heterogeneity in social and economic circumstances and policies and the capacity of centres to collect high-quality data with a modest budget.^12^

PURE’s data collection is described in detail elsewhere^12–14^; briefly, each country selected communities to include rural and urban populations, while ensuring feasibility of intended data collection methods (eg, processing blood samples) and long-term follow-up. Communities were defined as groups of people who reside within a specific geographic area and who were generally expected to have similar characteristics (eg, culture, socioeconomic status, use of amenities, goods and services). Existing administrative boundaries, such as village limits or postal code areas, or physical features (eg, area bounded by selected streets) were used to define urban communities, while rural communities were defined as villages or postal code areas located at least 50 km away from an urban centre. Households were selected to be broadly representative of their communities. All individuals within each selected household aged 35–70 years were eligible, and 1 50 447 individuals were enrolled. Each participant was interviewed using a standardised questionnaire, which collected data on lifestyle and behaviour, cardiovascular disease risk factors, health history, use of medications and social capital. Sitting blood pressure was measured twice by trained research assistants following a standardised procedure using a digital blood pressure measuring device (Omron HEM-757) and the average of these two measures was recorded. Community-level data, including availability and costs of medicines, and availability of public and private healthcare providers, were collected using the Environmental Profile of a Community’s Health instrument.^15^ Ethics committees at each centre approved the protocol, which has been published elsewhere,^12–14^ and all participants provided written informed consent.

In this analysis, only participants with hypertension were included, defined as those having an average systolic blood pressure (SBP) of at least 140 mm Hg, or an average diastolic blood pressure (DBP) of at least 90 mm Hg, or self-reporting as diagnosed with hypertension. We evaluated three binary outcomes of hypertension management: (1) detection, (2) treatment and (3) control among those with hypertension using the above definition. To isolate the stage in the pathway from detection to control where social capital has an effect, we also examined two conditional outcomes: (4) hypertension treatment and (5) control among those with detected hypertension. Detection was defined as having received a previous diagnosis of hypertension and to be using antihypertensive medication; treatment as using antihypertensive medication. Control was defined as participants with a history of hypertension whose average SBP and DBP was <140/90 mm Hg.

Following previous research, we measured bridging social capital in terms of participation in membership organisations and trust in people.^16^ Thus, our first bridging social capital variable, individual participation in local organisations, is denoted by *membership*, which takes the value one if individuals self-report as being members of any one of the following: self-help group, co-operative, social club, sports club or religious group; while it takes the value zero otherwise. It has been shown that social relationships between individuals sharing the same social identity increase well-being and informal insurance arrangements.^8^ Our second bridging variable, *trust in people,* is coded as one if individuals state they strongly believe that people are generally honest and want to help others, or if they strongly believe that others will reciprocate the respect shown to them, and zero otherwise. Informal mechanisms of insurance heavily rely on mutual trust.^17^

To capture linking social capital, we measure *trust in organisations,* based on how much individuals trust that organisations can be relied on for help in difficult situations. Again, we code this as one if individuals state that they can rely a great deal on civic or religious organisations for help, and zero otherwise.

Models included individual controls (sex, age, marital status, dummies for highest level of education obtained, household wealth quintile, urban-rural location, current or recent history of tobacco use, alcohol user, diabetes, depression, obesity, pregnancy, recent loss of employment), community controls (availability of any public health facility, any private health facility, availability of any antihypertensive medications at a retail pharmacy, number of different antihypertensive medication categories available at a retail pharmacy and potential proxies for community social capital^18^: presence of electric street lighting, presence of any traffic lights and completeness of paved roads) and country and year dummy variables that capture national and annual effects not otherwise directly measured. Online supplementary appendix file 1 provides full definitions of all model variables.

10.1136/bmjgh-2017-000443.supp1 Supplementary file 1

### Statistical modelling

To avoid issues with collinearity, we assessed the relationship between each social capital variable (eg, *membership*) and our hypertension management outcomes (eg, *detection*) using separate ordinary least-square models adjusting for community fixed effects (ie, community-specific slopes) as follows:

$$\begin{array}{ll} detection_{ic}-\bar{detection_{c}} & =\beta_{1}(membership_{ic}-\bar{membership_{c}})\\ & \;+\beta_{2}(I_{ic}-\bar{I_{c}})+\beta_{3}(C_{c}-\bar{C})\\ & \;+\beta_{4}(\gamma_{ic}-\bar{\gamma_{c}})+\beta_{5}\eta +(\varepsilon_{ic}-\bar{\varepsilon_{c}}) \end{array}$$

where the subscript *i* stands for the individual, and the subscript *c* for the community; *I* is a vector of explanatory variables at individual and household level, *C* is a vector of explanatory variables at community level, *γ* is a year dummy, *η* is a country dummy, *ε* is the disturbance term. Terms with a bar (eg, $\bar{detection_{c}}$) are the average observations over *i* individuals within community *c*.

We stratified the analysis into HICs and UMICs (ie, high-income countries), where we assumed that health systems will be stronger, and LMICs and LICs (ie, low-income countries), where we assumed that they will be weaker (see online supplementary appendix file 2). The stratification was based on World Bank income groups. We rejected stratifying based on existing measures of health system performance as they include health outcomes, thereby risking circularity. For each social capital variable, we tested for the equality of regression coefficients across country groups using the Chow test.^19^

## Results

Characteristics of the 61 229 hypertensive participants identified in the PURE sample are shown in table 1. About half (52%) of the participants identified in HICs and UMICs had their condition detected, compared with 43% in LMICs and LICs. Those who were being treated with antihypertensive medications ranged from 35% in LMICs and LICs up to 48% in UMICs and HICs. Hypertension control was poor in countries at all levels of economic development, averaging 11% in LMICs and LICs up to 17% in HICs and UMICs.

**Table 1:** Hypertension management characteristics of the sample by country and country income grouping

Country by country income group		No. of communities	No. of households	No. of hypertensive participants	Hypertensive participants					
					No. detected (%)		No. treated (%)		No. controlled (%)	
HIC and UMIC		250	21 903	25 429	13 155	(51.7)	12 231	(48.1)	4243	(16.7)
	Canada	75	3541	3908	2157	(55.2)	2112	(54.0)	968	(24.8)
	Sweden	24	1668	1923	696	(36.2)	608	(31.6)	169	(8.8)
	UAE	3	409	477	247	(51.8)	240	(50.3)	63	(13.2)
	Saudi Arabia	19	453	515	319	(61.9)	309	(60.0)	169	(32.8)
	Argentina	21	3148	3809	2073	(54.4)	1924	(50.5)	569	(14.9)
	Brazil	14	2480	2938	1896	(64.5)	1838	(62.6)	685	(23.3)
	Chile	5	1248	1530	890	(58.2)	798	(52.2)	332	(21.7)
	Malaysia	34	4842	5509	2648	(48.1)	2272	(41.2)	690	(12.5)
	Poland	4	1111	1368	739	(54.0)	706	(51.6)	151	(11.0)
	South Africa	12	1682	1857	580	(31.2)	616	(33.2)	117	(6.3)
	Turkey	39	1321	1595	910	(57.1)	808	(50.7)	330	(20.7)
LMIC and LIC		406	30 265	35 800	15 296	(42.7)	12 596	(35.2)	3830	(10.7)
	China	110	16 184	19 471	8114	(41.7)	6557	(33.7)	1556	(8.0)
	Philippines	2	678	855	466	(54.5)	394	(46.1)	115	(13.5)
	Colombia	60	2538	2817	1461	(51.9)	1309	(46.5)	484	(17.2)
	Iran	20	1411	1598	841	(52.6)	816	(51.1)	293	(18.3)
	OPT	39	583	591	347	(58.7)	345	(58.4)	130	(22.0)
	Bangladesh	56	897	1080	261	(24.2)	174	(16.1)	43	(4.0)
	India	95	6777	8090	3332	(41.2)	2708	(33.5)	1095	(13.5)
	Pakistan	4	373	435	206	(47.4)	162	(37.2)	76	(17.5)
	Zimbabwe	3	419	453	223	(49.2)	119	(26.3)	36	(7.9)
	Tanzania	17	405	410	45	(11.0)	12	(2.9)	2	(0.5)

HIC, high-income countries; LIC, low-income countries; LMIC, lower-middle-income countries; OPT, Occupied Palestinian Territories; UAE, United Arab Emirates; UMIC, upper-middle-income countries.

**Figure 1:**
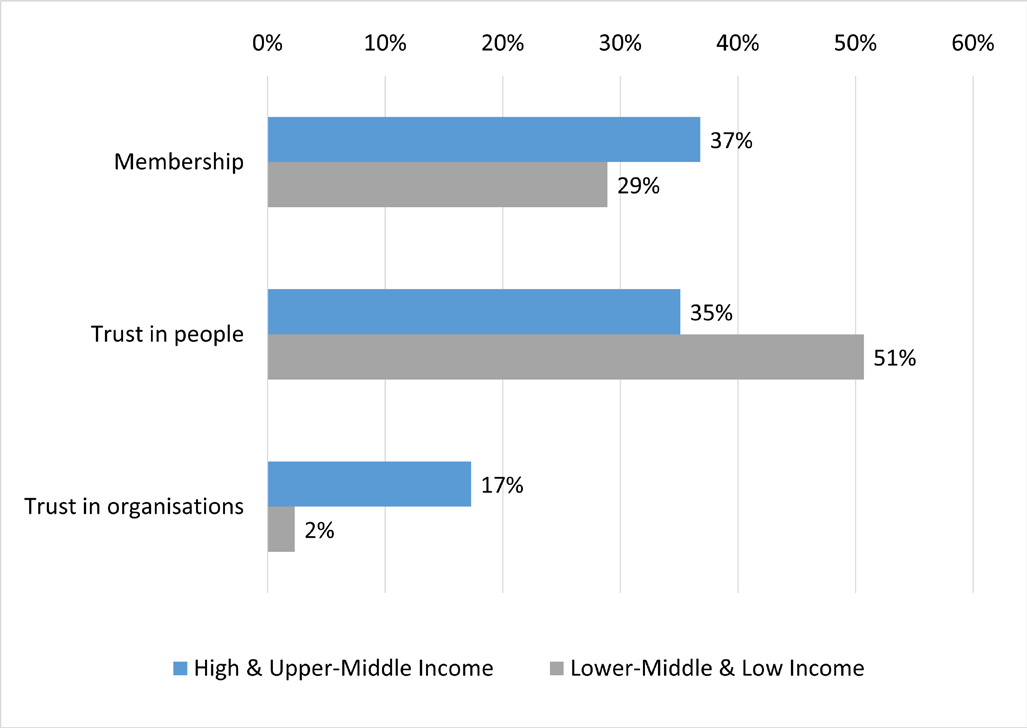
Levels of self-reported individual social capital by country income group.

Turning to social capital (figure 1), the proportion of hypertensive individuals who were members of a civic or religious organisation (bridging social capital) ranged from 29% in the LMIC and LIC group up to 37% in the HIC and UMIC group. However, few hypertensive individuals in each country grouping expressed a great deal of trust in such organisations (linking social capital), ranging from about 2% in LIC and LMICs up to 17% in HICs and UMICs; while many more reported a high degree of trust in other people (bridging social capital, from 35% in HICs and UMICs up to 51% in LMICs and LICs). Detailed summary statistics for all social capital, individual, household and community-level variables by country income group are shown in online supplementary appendix file 3.

### Associations of social capital with hypertension detection, treatment and control

Table 2 presents the estimated crude associations between the three social capital variables and each unconditional outcome along the hypertension management pathway from detection, through treatment with medication, to control in all hypertensive participants while controlling for age, sex and heterogeneity across countries (because of the very different levels of hypertension outcomes) using country dummy variables. This shows that *membership* in a social organisation is significantly associated with better detection, treatment and control in low-income countries (ie, LMICs and LICs), but only with detection and treatment in high-income countries (ie, HICs and UMICs). There is no significant association with the other two social capital variables.

**Table 2:** Crude associations between social capital variables and hypertension detection, treatment and control (among all hypertensive participants) by country income group, ordinary least-square estimates from model specification including only age, sex and country dummies as controls

Dimension of social capital	Hypertension detection		Hypertension treatment		Hypertension control	
	HIC and UMIC	LMIC and LIC	HIC and UMIC	LMIC and LIC	HIC and UMIC	LMIC and LIC
Membership						
Coefficient	0.020*	0.090**	0.016*	0.083**	0.002	0.050*
SE	0.008	0.012	0.008	0.013	0.005	0.010
Number of individuals	25 178	15 860	25 178	15 860	25 178	15 860
R^2^	0.087	0.085	0.103	0.105	0.045	0.025
P value for difference in coefficients†	0.0000		0.0000		0.0000	
Trust in people						
Coefficient	−0.004	0.001	−0.004	−0.011	0.002	−0.004
SE	0.008	0.010	0.008	0.009	0.006	0.005
Number of individuals	25 177	30 061	25 177	30 061	25 177	30 061
R^2^	0.087	0.061	0.102	0.080	0.045	0.027
P value for difference in coefficients†	0.7393		0.5181		0.3812	
Trust in organisations						
Coefficient	0.014	0.024	−0.004	0.010	0.003	0.000
SE	0.011	0.022	0.010	0.023	0.008	0.017
Number of individuals	24 937	29 858	24 937	29 858	24 937	29 858
R^2^	0.088	0.059	0.103	0.079	0.045	0.027
P value for difference in coefficients†	0.7711		0.6499		0.8325	

*P<0.05, **P<0.01, when testing hypothesis that coefficient is equal to 0.

†P value from Chow test for the equality of coefficients between HIC and UMIC vs LMIC and LIC groups.

HIC, high-income countries; LIC, low-income countries; LMIC, lower-middle-income countries; UMIC, upper-middle-income countries.

Table 3 then presents similar analysis, but controlling for a wide range of other variables, as noted above using the most conservative community fixed effect specification. In this fully adjusted model, it is only in low-income countries that *membership* in a social organisation is positively associated with the probability that hypertension will be detected, treated and controlled. The increased probabilities are modest, at 2.9% (95% CI 0.3% to 5.6%), 2.8% (95% CI 0.3% to 5.3%) and 2.7% (95% CI 0.9% to 4.6%), respectively. There was no significant association with *membership* in high-income countries for any of the three outcomes. There was significant heterogeneity in the association with *membership* across country income groups for hypertension control (P=0.0203). No notable associations or differences across country groups were observed between the other bridging social capital variable, *trust in people,* or the linking variable, *trust in organisations,* and any of the hypertension management outcomes (detection, treatment or control), regardless of country income grouping. Results from other less conservative models are shown in online supplementary appendix file 4.

**Table 3:** Fully adjusted associations between social capital variables and hypertension detection, treatment and control (among all hypertensive participants) by country income group, ordinary least-square estimates from model specification including individual controls †, community controls ‡ and community fixed effects

Dimension of social capital	Hypertension detection		Hypertension treatment		Hypertension control	
	HIC and UMIC	LMIC and LIC	HIC and UMIC	LMIC and LIC	HIC and UMIC	LMIC and LIC
Membership						
Coefficient	0.012	0.029*	0.008	0.028*	0.002	0.027**
SE	0.008	0.013	0.007	0.013	0.005	0.010
Number of individuals	22 381	10 467	22 381	10 467	22 381	10 467
R^2^	0.104	0.087	0.123	0.093	0.026	0.019
P value for difference in coefficients §	0.2631		0.1754		0.0203	
Trust in people						
Coefficient	0.004	−0.002	0.004	0.000	0.003	0.001
SE	0.008	0.009	0.007	0.008	0.006	0.004
Number of individuals	22 372	23 784	22 372	23 784	22 372	23 784
R^2^	0.103	0.058	0.123	0.062	0.026	0.012
P value for difference in coefficients §	0.6441		0.6927		0.7561	
Trust in organisations						
Coefficient	0.011	0.039	−0.002	0.000	0.008	0.011
SE	0.010	0.023	0.010	0.024	0.007	0.018
Number of individuals	22 222	23 756	22 222	23 756	22 222	23 756
R^2^	0.104	0.058	0.124	0.062	0.026	0.012
P value for difference in coefficients§	0.2696		0.9419		0.8686	

* P<0.05, **P<0.01, when testing hypothesis that coefficient is equal to 0.

†Individual and household controls include sex, age, marital status, dummies for highest level of education obtained, dummies for household wealth quintiles, urban-rural location, current or recent history of tobacco use, alcohol user, diabetes, depression, obesity, pregnancy, recent loss of employment, dummies for country and year of data collection.

‡Community controls include availability of any public health facility, any private health facility, availability of any antihypertensive medications at a retail pharmacy, number of different antihypertensive medication categories available at a retail pharmacy, presence of electric street lighting, presence of any traffic lights and completeness of paved roads.

§P value from Chow test for the equality of coefficients between HIC and UMIC vs LMIC and LIC groups.

HIC, high-income countries; LIC, low-income countries; LMIC, lower-middle-income countries; UMIC, upper-middle-income countries.

### Identifying the mechanisms by which social capital may influence hypertension management

If social capital improves adherence and access to treatment, over and above enhancing detection, then the small positive associations would persist after controlling for a detection effect. If, however, the only means of improvement were via increased detection, then there would be no residual effect on treatment and control after adjusting for detection. Table 4 shows the estimated conditional associations from the most conservative community fixed effect specification between the three social capital variables and hypertension treatment and control, using only those who have already had detected hypertension.

**Table 4:** Associations between social capital variables and hypertension detection, treatment and control (among participants aware of their hypertension) by country income group, ordinary least-square estimates from model specification including individual controls *, community controls† and community fixed effects

Dimension of social capital	Hypertension treatment		Hypertension control	
	HIC and UMIC	LMIC and LIC	HIC and UMIC	LMIC and LIC
Membership				
Coefficient	−0.002	0.014	−0.001	0.032
SE	0.007	0.012	0.009	0.016
Observations	11 683	4912	11 683	4912
R^2^	0.040	0.042	0.019	0.015
P value for difference in coefficients‡	0.2425		0.0728	
Trust in people				
Coefficient	0.005	−0.007	0.007	0.004
SE	0.007	0.009	0.010	0.008
Observations	11 679	10 516	11 679	10 516
R^2^	0.040	0.019	0.019	0.009
P value for difference in coefficients‡	0.2820		0.7960	
Trust in organisations				
Coefficient	−0.014	−0.037	0.008	−0.002
SE	0.009	0.029	0.012	0.029
Observations	11 614	10 494	11 614	10 494
R^2^	0.041	0.020	0.019	0.009
P value for difference in coefficients‡	0.4533		0.7474	

*Individual and household controls include sex, age, marital status, dummies for highest level of education obtained, dummies for household wealth quintiles, urban-rural location, current or recent history of tobacco use, alcohol user, diabetes, depression, obesity, pregnancy, recent loss of employment, dummies for country and year of data collection.

†Community controls include availability of any public health facility, any private health facility, availability of any antihypertensive medications at a retail pharmacy, number of different antihypertensive medication categories available at a retail pharmacy, presence of electric street lighting, presence of any traffic lights and completeness of paved roads.

‡P value from Chow test for the equality of coefficients between HIC and UMIC vs LMIC and LIC groups.

HIC, high-income countries; LIC, low-income countries; LMIC, lower-middle-income countries; UMIC, upper-middle-income countries.

In this group, the association between *membership* and treatment seen in the larger sample of hypertensive participants was no longer present (table 4). However, among those whose hypertension had been detected, *membership* increased the likelihood of control by an average of 3.2%, although the 95% CI just included zero (0.0%–6.5%) and only in low-income countries. Heterogeneity tests confirmed that this association with *membership* was present among those only in the low-income countries.

Finally, we limited the analysis to low-income countries and stratified it by urban and rural setting (table 5). The association between *membership* and hypertension control remained in both settings although, while the size of the coefficient was similar to that in the overall sample, in rural settings it was of borderline statistical significance (P=0.064). In this case, *trust in organisations* was significantly associated with improved detection, but only in rural areas where it is to be expected that health systems may be weaker.

**Table 5:** Associations between social capital variables and hypertension detection, treatment and control (among all hypertensive participants) in LMICs and LICs only by urban-rural location, ordinary least-square estimates from model specification including individual controls†, community controls‡ and community fixed effects

Dimension of social capital	Hypertension detection		Hypertension treatment		Hypertension control	
	Urban	Rural	Urban	Rural	Urban	Rural
Membership						
Coefficient	0.024	0.033	0.028	0.024	0.025*	0.030
SE	(0.018)	(0.020)	(0.017)	(0.020)	(0.012)	(0.016)
Observations	6163	4304	6163	4304	6163	4304
R^2^	0.083	0.100	0.091	0.096	0.019	0.023
P value for difference in coefficients§	0.7368		0.8946		0.7694	
Trust in people						
Coefficient	0.000	−0.005	0.007	−0.011	0.006	−0.007
SE	(0.011)	(0.013)	(0.011)	(0.011)	(0.006)	(0.005)
Observations	13 330	10 454	13 330	10 454	13 330	10 454
R^2^	0.054	0.067	0.059	0.069	0.012	0.014
P value for difference in coefficients§	0.7560		0.2363		0.0936	
Trust in organisations						
Coefficient	0.019	0.057*	0.007	−0.007	0.016	0.003
SE	(0.034)	(0.029)	(0.039)	(0.028)	(0.032)	(0.016)
Observations	13 311	10 445	13 311	10 445	13 311	10 445
R^2^	0.054	0.067	0.059	0.069	0.012	0.014
P value for difference in coefficients §	0.3873		0.7619		0.7097	

*P<0.05, when testing hypothesis that coefficient is equal to 0.

†Individual and household controls include sex, age, marital status, dummies for highest level of education obtained, dummies for household wealth quintiles, urban-rural location, current or recent history of tobacco use, alcohol user, diabetes, depression, obesity, pregnancy, recent loss of employment, dummies for country and year of data collection.

‡Community controls include availability of any public health facility, any private health facility, availability of any antihypertensive medications at a retail pharmacy, number of different antihypertensive medication categories available at a retail pharmacy, presence of electric street lighting, presence of any traffic lights and completeness of paved roads.

§P value from Chow test for the equality of coefficients between HIC and UMIC vs LMIC and LIC groups.

HIC, high-income countries; LIC, low-income countries; LMIC, lower-middle-income countries; UMIC, upper-middle-income countries.

### Robustness checks and model validity

We assessed the robustness of our findings using a series of model specifications, including only individual-level controls as well as individual-level plus community-level controls in both the full sample of all hypertensive participants (online supplementary appendix file 4) and the subsample of those with their hypertension detected (online supplementary appendix file 5). None of the results were qualitatively changed. However, the estimated associations from the specifications including only individual controls, and individual plus community controls tended to be of a greater magnitude, consistent with potential unobserved community confounding factors.

The estimated associations of our covariates were in the anticipated direction, adding to the validity of our findings. The full model results, including those for the full range of control variables, are provided in online supplementary appendix file 6. Briefly, the statistically significant positive associations of non-modifiable control variables, such as sex, age and comorbidities (eg, depression and diabetes) on hypertension management outcomes were consistent both across country income grouping and model specification.

10.1136/bmjgh-2017-000443.supp2 Supplementary file 2

## Discussion

Although the association between social capital and health status has been examined extensively elsewhere, this is the first cross-regional study that examines its association with the management of common non-communicable diseases (NCDs) or their risk factors. In addition, very few studies on social capital and health have included low-income and middle-income countries, which now account for the majority of the NCD burden.

Our findings differ between countries at different income levels, which we take as a proxy for health system strength: we find no detectable association of bridging or linking social capital with hypertension management in high-income countries. This is unsurprising as they have effective institutions and procedures that leave little scope for social networks to bring additional benefit in obtaining care. This finding is consistent with other research showing that the health benefits associated with social capital are less in countries with better functioning employment protection systems.^20^ However within high-income countries, social capital may still have an important function for groups with poorer access to care. While such analysis is beyond the remit of this paper, the question does warrant further research.

In low-income countries, which we consider to be more likely to have weaker systems, we find that individual-level social capital is associated with an increase in the likelihood of having hypertension detected, treated and controlled, although modestly, linked to bridging social capital, as measured by participation in civic and religious organisations. The observed positive association with treatment is explained entirely by increased detection, as indicated by the conditional analysis among known hypertensives. Linking social capital is also associated with increased detection, although only in rural areas of low-income countries, where we would expect health systems to be especially weak. Importantly, the coefficients are consistently in the expected direction. These associations are small, as expected. The quality of hypertension management is largely a reflection of the wider health system, including affordability and availability of medicines, as shown in other analyses using PURE data,^21^ as well as of personal characteristics such as education and income.^22^ All of these are controlled for in our models, so any observed association is in addition to these factors. Our model also adjusts for behaviours such as smoking as they may reveal unobserved factors such as time preferences.^23^

Our use of community fixed effects models removes many unobserved factors at that level, including community-level social capital, which we were unable to measure directly. This could bring further benefits, although other studies have found its impact to be limited.^3 17^ Consequently, our estimates must be considered somewhat conservative.

Before interpreting our findings, we note several limitations. First, while we have directly adjusted for potential confounding factors, there may be residual endogeneity, as with any observational study. A lack of available data and the cross-sectional design limit our ability to establish causality. Second, our binary measures of individual-level social capital are blunt measures of a complex concept and do not take into account the intensity of social participation, for example. The PURE study was not designed to measure social capital as a primary aim, although the tool employed questions adapted from previous social capital studies, and collected information covering all social capital dimensions. We also cannot rule out the potential effect of healthy volunteer selection bias; and all social capital variables were not collected in every country (see online supplementary appendix file 3). Finally, our study did not include a measure of bonding social capital, which may have even more substantial and positive impacts on hypertension management. Data on whether family members could be counted on in difficult situations was collected to inform this dimension of social capital only in a subset of countries (Saudi Arabia, Turkey, Philippines, Bangladesh, Zimbabwe, Tanzania). Supplemental analysis showed that bonding social capital did not modify the association of the bridging and linking forms with hypertension management, which were the main foci of this study.

Several possible mechanisms may be involved. Social capital may facilitate exchange of information on the quality of services and informal mechanisms of obtaining care, thereby increasing detection. Trust in civic and religious organisations may spill over to trust in the health system, thus increasing the likelihood of seeking healthcare, leading to detection, and regular monitoring once diagnosed.^24^ These outcomes could also arise from the support and influence of the trust invested in people and trustworthy interpersonal networks that result from increased social participation. For example, organisational membership could bring individuals into contact with others with similar common conditions, thereby enhancing knowledge of hypertension and how to manage it, making it easier to access care, and providing informal support that motivates individuals to seek regular care, adhere to medication and maintain long-term lifestyle changes. Evidence from other studies provides some support for such mechanisms. For example, a Swedish study found some evidence for an association between low social participation and poor adherence to antihypertensive treatment^25^, while a study of British patients with chronic heart disease or diabetes found that social networks provided a substitute for formal care, improving self-management outcomes and reducing healthcare costs.^26^ Better adherence to lifestyle modifications was also observed in a longitudinal study of British smokers, where trust and social participation were positively associated with smoking cessation.^27^ Clearly, further research, particularly qualitative investigation, is needed to understand better the complex mechanisms at work.

Our findings suggest that, in low-income countries, social capital increases the probability of treatment by enabling more people with hypertension to be diagnosed. In other words, if we consider the entire hypertension management pathway, being a member and having trust in social organisations may encourage people to seek care, which in turn may increase the probability that they will be diagnosed. This is unsurprising; the decision to seek care is made by the individual, although subject to a variety of facilitators and constraints. It is the health professional who, after treatment has been sought, is primarily responsible for the decision to recommend treatment and he or she will largely be uninfluenced by the individual’s social capital. However, the individual with hypertension must also accept treatment, a decision likely to be influenced by the availability and affordability of medicines.^21^ Thus, the absence of an effect may be because the type of support provided by social networks is not primarily financial. Because antihypertensive medications are unaffordable for many in low-income and middle-income country settings and typically require large out-of-pocket payments,^21^ such networks provide little help in this regard, at least in relation to long-term financial commitments. Social capital does, however, seem to play a modest role in improving control. Although a 3% improvement in hypertension detection, treatment and control may seem unimportant, given that hypertension affects millions in low-income countries, with less than half being detected, about one-fourth being treated and only about one-sixth controlled, such an improvement at all of the points along the treatment pathway represents improved management of several tens of millions of people, which should translate into the prevention of a several tens of thousands of cardiovascular events each year. In addition, the benefits of social capital may extend to other conditions that require detection and long-term treatment such as diabetes, so the cumulative impact of enhanced social capital across multiple conditions could be substantial.

Our results have potential implications for policy. They reinforce the call by Woolcock and Narayan to consider social capital as a component of health systems reform in low-income countries,^28^ reiterating the importance of embedding change in existing social structures and strengthening trust in institutions. There are several opportunities to do this, drawing on evidence from research on community-driven development that social capital can be strengthened.^29^ First, reforms should incorporate participatory processes, and especially those that transcend social and other divides. Our findings and those from the studies cited above highlight the potential role that social networks play, bridging such divides, to benefit the management of long-term conditions like hypertension. They also point towards the potential for community and network-centred approaches to support health literacy and chronic illness management, which some have suggested may be more appropriate for engaging people in socially and economically deprived contexts.^5^ Second, reforms should include measures that enhance trust in public health facilities, including measures to strengthen the quality and acceptability of care, and information disclosure and other efforts to promote transparency. Third, they should explore opportunities for strengthening the exchange of information on services and how to access them. Of course, all these are justified in themselves, but these findings point to a non-trivial health benefit.

In conclusion, greater bridging and linking social capital may help individuals with hypertension to access and benefit from healthcare where health systems are weak. It is not an alternative to strengthening health systems, but it may play an important role in improving management. Health system reform should recognise this, where possible, adopting measures that increase social capital (evaluating its effects, given the scarcity of research), but at a minimum avoid policies that erode it by damaging trust and fragmenting networks.
