# Supplementary material for: Does greater individual social capital improve the management of hypertension? Cross-national analysis of 61 229 individuals in 21 countries
Source: BMJ Glob Health. 2017 Dec 17;2(4):e000443. doi: 10.1136/bmjgh-2017-000443 (PMC5759715; doi:10.1136/bmjgh-2017-000443)
Supplement: Supplementary file 2 [file bmjgh-2017-000443supp002.pdf]

Effect of membership of any social organisation on hypertension detection, treatment and control among ALL hypertensives

Hypertension detection

VARIABLES	OLS+individual controls HIC & UMIC	OLS+individual controls LMIC & LIC	OLS+individual+ community controls HIC & UMIC	OLS+individual+ community controls LMIC & LIC	OLS+individual+ community controls + community fixed effects HIC & UMIC	OLS+individual+ community controls + community fixed effects LMIC & LIC
Membership	0.012	0.058***	0.011	0.058***	0.012	0.029**
	(0.008)	(0.013)	(0.008)	(0.014)	(0.008)	(0.013)
Female	0.085***	0.146***	0.085***	0.139***	0.084***	0.119***
	(0.008)	(0.015)	(0.008)	(0.016)	(0.008)	(0.016)
Age	0.030***	0.031***	0.031***	0.035***	0.030***	0.031***
	(0.004)	(0.005)	(0.004)	(0.005)	(0.004)	(0.005)
Age squared	-0.000***	-0.000***	-0.000***	-0.000***	-0.000***	-0.000***
	(0.000)	(0.000)	(0.000)	(0.000)	(0.000)	(0.000)
Urban	0.006	0.020	0.014	0.015	-0.079	0.349***
	(0.011)	(0.021)	(0.013)	(0.026)	(0.064)	(0.061)
Married	0.005	0.024*	0.005	0.029**	0.000	0.038**
	(0.007)	(0.013)	(0.007)	(0.015)	(0.007)	(0.015)
Secondary education	-0.017*	0.068***	-0.017*	0.060***	-0.014	0.043***
	(0.009)	(0.016)	(0.009)	(0.016)	(0.010)	(0.016)
Tertiary education	-0.033***	0.086***	-0.033**	0.074***	-0.028**	0.063***
	(0.012)	(0.021)	(0.013)	(0.022)	(0.013)	(0.021)
Wealth quintile 2	0.045	0.052***	0.061**	0.053***	0.030	0.020
	(0.031)	(0.015)	(0.028)	(0.017)	(0.028)	(0.016)
Wealth quintile 3	0.079*	0.094***	0.097***	0.089***	0.059*	0.058***
	(0.040)	(0.017)	(0.037)	(0.018)	(0.033)	(0.019)
Wealth quintile 4	0.072*	0.106***	0.087**	0.097***	0.057*	0.069***
	(0.040)	(0.019)	(0.037)	(0.019)	(0.033)	(0.022)
Wealth quintile 5	0.063	0.103***	0.081**	0.096***	0.057*	0.067**
	(0.041)	(0.025)	(0.037)	(0.027)	(0.034)	(0.027)
Tobacco user	0.008	-0.010	0.006	-0.011	0.004	-0.003
	(0.009)	(0.013)	(0.009)	(0.014)	(0.009)	(0.014)
Alcohol user	-0.041***	0.028**	-0.042***	0.031**	-0.033***	0.007
	(0.010)	(0.013)	(0.011)	(0.015)	(0.012)	(0.015)
Diabetes	0.273***	0.235***	0.266***	0.214***	0.261***	0.196***
	(0.014)	(0.014)	(0.014)	(0.014)	(0.014)	(0.014)
Depressed	0.070***	0.070***	0.075***	0.077***	0.076***	0.087***
	(0.007)	(0.012)	(0.007)	(0.012)	(0.008)	(0.011)
Pregnant	0.037	0.031	0.065	0.058	0.060	0.072
	(0.040)	(0.055)	(0.040)	(0.061)	(0.041)	(0.060)
Recently lost job	0.003	-0.030	0.006	-0.033	0.002	-0.032
	(0.017)	(0.023)	(0.017)	(0.024)	(0.017)	(0.023)
Obese	0.094***	0.083***	0.095***	0.085***	0.091***	0.078***
	(0.007)	(0.012)	(0.008)	(0.013)	(0.008)	(0.013)
year5	-0.112***	-0.132***	-0.154**	-0.150***	0.052	-0.078
	(0.040)	(0.044)	(0.073)	(0.046)	(0.049)	(0.093)
year6	-0.091***	-0.149***	-0.134**	-0.166***	-0.042	-0.079
	(0.032)	(0.045)	(0.053)	(0.046)	(0.030)	(0.100)
year7	-0.071**	-0.190***	-0.114**	-0.211***	-0.031	-0.134
	(0.033)	(0.044)	(0.053)	(0.047)	(0.028)	(0.101)
year8	-0.071**	-0.117**	-0.120**	-0.124**	-0.019	-0.096
	(0.032)	(0.049)	(0.052)	(0.053)	(0.025)	(0.111)
year9	-0.055*	-0.111**	-0.108**	-0.133**	-0.005	-0.247**
	(0.031)	(0.052)	(0.050)	(0.055)	(0.019)	(0.116)
country5			0.067			
			(0.065)			
country7	0.200***		0.284***			
	(0.024)		(0.063)			
country8	-0.024		0.054			
	(0.026)		(0.064)			
country9	0.100***		0.186***			
	(0.028)		(0.066)			
country11	0.107***		0.198***			
	(0.025)		(0.065)			
country12	0.091***		0.174***			

Hypertension treatment

VARIABLES	OLS+individual controls HIC & UMIC	OLS+individual controls LMIC & LIC	OLS+individual+ community controls HIC & UMIC	OLS+individual+ community controls LMIC & LIC	OLS+individual+ community controls + community fixed effects HIC & UMIC	OLS+individual+ community controls + community fixed effects LMIC & LIC
Membership	0.009	0.049***	0.008	0.048***	0.008	0.028**
	(0.007)	(0.013)	(0.007)	(0.014)	(0.007)	(0.013)
Female	0.083***	0.139***	0.082***	0.143***	0.083***	0.120***
	(0.008)	(0.014)	(0.008)	(0.016)	(0.008)	(0.015)
Age	0.029***	0.028***	0.029***	0.031***	0.029***	0.027***
	(0.004)	(0.004)	(0.004)	(0.005)	(0.004)	(0.005)
Age squared	-0.000***	-0.000***	-0.000***	-0.000***	-0.000***	-0.000***
	(0.000)	(0.000)	(0.000)	(0.000)	(0.000)	(0.000)
Urban	-0.001	0.021	-0.000	0.017	-0.151**	0.305***
	(0.012)	(0.021)	(0.014)	(0.026)	(0.058)	(0.063)
Married	0.017**	0.011	0.017**	0.013	0.014*	0.024*
	(0.008)	(0.012)	(0.008)	(0.013)	(0.008)	(0.013)
Secondary education	0.002	0.074***	0.005	0.064***	0.009	0.042***
	(0.009)	(0.014)	(0.010)	(0.015)	(0.009)	(0.013)
Tertiary education	-0.016	0.097***	-0.011	0.086***	-0.008	0.069***
	(0.012)	(0.017)	(0.013)	(0.017)	(0.012)	(0.015)
Wealth quintile 2	0.029	0.052***	0.037	0.045***	0.008	0.023*
	(0.025)	(0.014)	(0.025)	(0.015)	(0.024)	(0.013)
Wealth quintile 3	0.076**	0.086***	0.086***	0.072***	0.048	0.054***
	(0.032)	(0.017)	(0.033)	(0.019)	(0.029)	(0.018)
Wealth quintile 4	0.064**	0.119***	0.073**	0.098***	0.037	0.081***
	(0.031)	(0.021)	(0.031)	(0.023)	(0.028)	(0.023)
Wealth quintile 5	0.057*	0.118***	0.067**	0.100***	0.035	0.079***
	(0.031)	(0.028)	(0.031)	(0.032)	(0.028)	(0.029)
Tobacco user	0.003	0.000	0.001	0.004	0.000	0.008
	(0.008)	(0.012)	(0.008)	(0.013)	(0.008)	(0.013)
Alcohol user	-0.054***	0.017	-0.055***	0.021	-0.047***	-0.001
	(0.009)	(0.014)	(0.010)	(0.015)	(0.011)	(0.014)
Diabetes	0.279***	0.225***	0.276***	0.215***	0.272***	0.198***
	(0.013)	(0.013)	(0.014)	(0.014)	(0.014)	(0.015)
Depressed	0.052***	0.057***	0.055***	0.062***	0.059***	0.074***
	(0.008)	(0.011)	(0.008)	(0.011)	(0.008)	(0.010)
Pregnant	0.067	0.055	0.081*	0.065	0.076*	0.083
	(0.042)	(0.053)	(0.043)	(0.060)	(0.043)	(0.056)
Recently lost job	-0.009	-0.032	-0.008	-0.037	-0.007	-0.035
	(0.014)	(0.023)	(0.015)	(0.024)	(0.015)	(0.024)
Obese	0.097***	0.070***	0.098***	0.069***	0.096***	0.066***
	(0.007)	(0.012)	(0.007)	(0.013)	(0.007)	(0.013)
year5	0.064	-0.103**	0.043	-0.124**	0.055	-0.077
	(0.046)	(0.049)	(0.085)	(0.050)	(0.067)	(0.104)
year6	-0.025	-0.085*	-0.066	-0.101*	-0.048	-0.005
	(0.032)	(0.049)	(0.061)	(0.051)	(0.056)	(0.117)
year7	0.012	-0.142***	-0.029	-0.161***	-0.016	-0.050
	(0.032)	(0.048)	(0.060)	(0.051)	(0.055)	(0.117)
year8	0.016	-0.056	-0.030	-0.071	0.002	0.012
	(0.031)	(0.053)	(0.060)	(0.057)	(0.054)	(0.126)
year9	0.011	-0.087	-0.036	-0.105*	0.003	-0.151
	(0.028)	(0.055)	(0.057)	(0.059)	(0.052)	(0.162)
country5			0.055			
			(0.050)			
country7	0.201***		0.267***			
	(0.029)		(0.048)			
country8	-0.052*		0.002			
	(0.030)		(0.047)			
country9	0.061		0.124**			
	(0.038)		(0.054)			
country11	0.113***		0.179***			
	(0.029)		(0.048)			
country12	0.082***		0.147***			

Hypertension control

VARIABLES	OLS+individual controls HIC & UMIC	OLS+individual controls LMIC & LIC	OLS+individual+ community controls HIC & UMIC	OLS+individual+ community controls LMIC & LIC	OLS+individual+ community controls + community fixed effects HIC & UMIC	OLS+individual+ community controls + community fixed effects LMIC & LIC
Membership	-0.001	0.036***	-0.000	0.039***	0.002	0.027***
	(0.005)	(0.009)	(0.005)	(0.010)	(0.005)	(0.010)
Female	0.072***	0.062***	0.072***	0.065***	0.072***	0.053***
	(0.006)	(0.009)	(0.006)	(0.010)	(0.006)	(0.009)
Age	0.013***	0.014***	0.013***	0.015***	0.013***	0.012***
	(0.003)	(0.003)	(0.003)	(0.004)	(0.003)	(0.004)
Age squared	-0.000***	-0.000***	-0.000***	-0.000***	-0.000***	-0.000***
	(0.000)	(0.000)	(0.000)	(0.000)	(0.000)	(0.000)
Urban	0.012	0.015	0.015	0.017	-0.067	0.048*
	(0.009)	(0.014)	(0.011)	(0.017)	(0.094)	(0.028)
Married	0.002	0.010	0.000	0.009	-0.001	0.019*
	(0.006)	(0.009)	(0.006)	(0.010)	(0.006)	(0.010)
Secondary education	0.002	0.042***	0.001	0.042***	0.002	0.025**
	(0.007)	(0.009)	(0.008)	(0.010)	(0.008)	(0.010)
Tertiary education	0.015	0.072***	0.017	0.073***	0.019*	0.059***
	(0.011)	(0.014)	(0.011)	(0.015)	(0.011)	(0.014)
Wealth quintile 2	0.016	0.023**	0.016	0.020*	0.011	0.007
	(0.011)	(0.010)	(0.013)	(0.011)	(0.014)	(0.010)
Wealth quintile 3	0.034**	0.043***	0.033*	0.037***	0.018	0.023*
	(0.015)	(0.013)	(0.017)	(0.014)	(0.016)	(0.013)
Wealth quintile 4	0.048***	0.056***	0.046***	0.039**	0.033**	0.026*
	(0.015)	(0.018)	(0.016)	(0.016)	(0.016)	(0.015)
Wealth quintile 5	0.038**	0.058**	0.036**	0.040*	0.024	0.024
	(0.016)	(0.022)	(0.017)	(0.022)	(0.017)	(0.022)
Tobacco user	0.016***	0.017**	0.018***	0.020*	0.017***	0.018*
	(0.005)	(0.009)	(0.006)	(0.010)	(0.006)	(0.010)
Alcohol user	-0.032***	0.001	-0.033***	0.006	-0.028***	-0.006
	(0.006)	(0.011)	(0.006)	(0.012)	(0.006)	(0.011)
Diabetes	0.092***	0.079***	0.080***	0.077***	0.076***	0.071***
	(0.010)	(0.010)	(0.010)	(0.011)	(0.010)	(0.011)
Depressed	0.043***	0.026***	0.044***	0.026***	0.047***	0.031***
	(0.006)	(0.009)	(0.007)	(0.009)	(0.006)	(0.009)
Pregnant	0.048	0.089*	0.057	0.094	0.052	0.082
	(0.037)	(0.053)	(0.041)	(0.060)	(0.042)	(0.059)
Recently lost job	0.010	-0.036***	0.010	-0.042***	0.010	-0.034**
	(0.012)	(0.012)	(0.013)	(0.013)	(0.013)	(0.013)
Obese	0.006	0.004	0.005	0.003	0.004	0.001
	(0.005)	(0.008)	(0.006)	(0.009)	(0.005)	(0.008)
year5	-0.008	-0.125***	-0.000	-0.132***	0.065	-0.045
	(0.012)	(0.036)	(0.028)	(0.035)	(0.048)	(0.065)
year6	-0.072***	-0.107***	-0.069**	-0.109***	-0.043	0.004
	(0.023)	(0.035)	(0.033)	(0.034)	(0.037)	(0.068)
year7	-0.048**	-0.138***	-0.046	-0.147***	-0.027	-0.002
	(0.021)	(0.037)	(0.032)	(0.035)	(0.035)	(0.067)
year8	-0.056***	-0.027	-0.055*	-0.025	-0.038	0.069
	(0.021)	(0.042)	(0.032)	(0.042)	(0.034)	(0.078)
year9	-0.040**	-0.064	-0.043	-0.069	-0.037	-0.010
	(0.018)	(0.043)	(0.029)	(0.043)	(0.031)	(0.095)
country5			0.055*			
			(0.029)			
country7	0.125***		0.178***			
	(0.021)		(0.029)			
country8	0.001		0.057**			
	(0.019)		(0.027)			
country9	0.105***		0.159***			
	(0.028)		(0.034)			
country11	0.142***		0.198***			
	(0.019)		(0.027)			
country12	0.051***		0.106***			

	(0.025)		(0.062)				(0.030)		(0.046)				(0.019)		(0.026)			
country13	0.104**		0.184***				0.093**		0.151***				0.006		0.060*			
	(0.043)		(0.070)				(0.046)		(0.055)				(0.027)		(0.034)			
country14	0.017		0.110*				-0.032		0.035				0.008		0.067***			
	(0.025)		(0.058)				(0.030)		(0.042)				(0.021)		(0.022)			
country16	0.035		0.118*				-0.000		0.060				0.065**		0.125***			
	(0.029)		(0.064)				(0.033)		(0.046)				(0.025)		(0.030)			
Public health facility available			0.064**	-0.029					0.079*	-0.034					0.032**	-0.047**		
			(0.028)	(0.062)					(0.043)	(0.029)					(0.013)	(0.022)		
Private health facility available			0.009	-0.040					0.012	-0.011					-0.007	-0.006		
			(0.014)	(0.040)					(0.014)	(0.037)					(0.013)	(0.028)		
Any antihypertensive drug available			-0.010	0.169**					-0.017	0.191***					-0.002	0.105***		
			(0.037)	(0.066)					(0.039)	(0.069)					(0.030)	(0.040)		
Number of antihypertensive drug available			-0.002	-0.008					-0.001	-0.011					0.001	-0.014***		
			(0.004)	(0.008)					(0.004)	(0.007)					(0.004)	(0.005)		
Electric street lighting present			0.040	0.050					0.041	0.076**					0.020	0.041**		
			(0.024)	(0.039)					(0.028)	(0.036)					(0.020)	(0.017)		
Traffic lights present			-0.022	-0.003					-0.013	0.002					0.002	0.020		
			(0.013)	(0.024)					(0.015)	(0.025)					(0.010)	(0.017)		
Completeness of road paving			-0.014	0.031					-0.006	0.032					-0.009	0.000		
			(0.019)	(0.020)					(0.019)	(0.019)					(0.015)	(0.012)		
year13	-0.058	-0.026					-0.012	-0.025					-0.051	-0.013				
	(0.077)	(0.055)					(0.075)	(0.023)					(0.072)	(0.017)				
year14	-0.039	0.135**					-0.004	0.151***					-0.149**	-0.138***				
	(0.082)	(0.054)					(0.090)	(0.027)					(0.068)	(0.020)				
country3	-0.074						-0.056						-0.058**					
	(0.063)						(0.049)						(0.026)					
country20	0.099						0.139*						0.204***					
	(0.079)						(0.083)						(0.071)					
year2		-0.018		-0.033		0.024		0.018		0.004		0.052		-0.013		-0.013		0.017
		(0.047)		(0.045)		(0.034)		(0.049)		(0.047)		(0.042)		(0.038)		(0.035)		(0.032)
year3		-0.153***		-0.178***		-0.025		-0.137*		-0.164**		-0.044		-0.115***		-0.121***		-0.029
		(0.055)		(0.056)		(0.080)		(0.074)		(0.073)		(0.078)		(0.043)		(0.042)		(0.063)
year4		-0.107*		-0.127*		-0.087		-0.084		-0.105		-0.068		-0.084*		-0.085*		-0.023
		(0.062)		(0.066)		(0.077)		(0.066)		(0.067)		(0.082)		(0.045)		(0.044)		(0.059)
year10		-0.030		-0.097		-0.563***		-0.052		-0.131*		-0.353**		-0.036		-0.125**		-0.081
		(0.053)		(0.089)		(0.118)		(0.058)		(0.073)		(0.163)		(0.045)		(0.052)		(0.096)
year11		-0.061		-0.130		-0.603***		-0.063		-0.145**		-0.375**		-0.059		-0.151***		-0.112
		(0.054)		(0.089)		(0.119)		(0.059)		(0.074)		(0.163)		(0.045)		(0.053)		(0.095)
country4		0.076***		0.041				0.086***		0.050*				0.015		0.005		
		(0.027)		(0.027)				(0.026)		(0.026)				(0.019)		(0.021)		
country6		0.056		0.038				-0.105***		-0.117***				-0.048**		-0.054**		
		(0.040)		(0.041)				(0.027)		(0.034)				(0.019)		(0.023)		
country10		0.013		-0.010				0.059**		0.040				-0.025		-0.021		
		(0.032)		(0.032)				(0.030)		(0.029)				(0.025)		(0.025)		
country15		-0.041						-0.084**						-0.123***				
		(0.036)						(0.034)						(0.028)				
country17		-0.005		-0.007				-0.005		-0.009				-0.026		-0.038		
		(0.035)		(0.049)				(0.039)		(0.043)				(0.031)		(0.033)		
country18		-0.371***						-0.326***						-0.177***				
		(0.056)						(0.048)						(0.038)				
country21		-0.016						0.003						-0.033				
		(0.067)						(0.050)						(0.039)				
Constant	-0.740***	-0.750***	-0.850***	-1.009***	-0.653***	-0.900***	-0.890***	-0.787***	-0.985***	-1.056***	-0.687***	-0.884***	-0.355***	-0.282***	-0.424***	-0.323***	-0.236*	-0.315***
	(0.116)	(0.128)	(0.122)	(0.176)	(0.131)	(0.156)	(0.102)	(0.126)	(0.139)	(0.158)	(0.132)	(0.148)	(0.091)	(0.094)	(0.092)	(0.118)	(0.129)	(0.110)
Observations	23,879	12,010	22,381	10,467	22,381	10,467	23,879	12,010	22,381	10,467	22,381	10,467	23,879	12,010	22,381	10,467	22,381	10,467
R-squared	0.142	0.150	0.139	0.132	0.104	0.087	0.158	0.170	0.157	0.153	0.123	0.093	0.058	0.058	0.054	0.052	0.026	0.019
Number of epochid					211	168					211	168					211	168

Robust standard errors in parentheses
*** p<0.01, ** p<0.05, * p<0.1

Effect of trust in people on hypertension detection, treatment and control among ALL hypertensives

	Hypertension detection						Hypertension treatment						Hypertension control					
	OLS+individual controls		OLS+individual+ community controls		OLS+individual+ community controls + community fixed effects		OLS+individual controls		OLS+individual+ community controls		OLS+individual+ community controls + community fixed effects		OLS+individual controls		OLS+individual+ community controls		OLS+individual+ community controls + community fixed effects	
VARIABLES	HIC & UMIC	LMIC & LIC	HIC & UMIC	LMIC & LIC	HIC & UMIC	LMIC & LIC	HIC & UMIC	LMIC & LIC	HIC & UMIC	LMIC & LIC	HIC & UMIC	LMIC & LIC	HIC & UMIC	LMIC & LIC	HIC & UMIC	LMIC & LIC	HIC & UMIC	LMIC & LIC
Trust in people	0.000	0.009	0.000	0.007	0.004	-0.002	0.003	0.003	-0.000	0.003	0.004	-0.000	0.005	0.002	0.005	0.002	0.003	0.001
	(0.008)	(0.009)	(0.008)	(0.009)	(0.008)	(0.009)	(0.008)	(0.008)	(0.008)	(0.009)	(0.007)	(0.008)	(0.006)	(0.005)	(0.006)	(0.005)	(0.006)	(0.004)
Female	0.086***	0.092***	0.085***	0.091***	0.085***	0.078***	0.083***	0.093***	0.083***	0.097***	0.083***	0.080***	0.072***	0.031***	0.072***	0.031***	0.072***	0.023***
	(0.008)	(0.012)	(0.008)	(0.013)	(0.008)	(0.010)	(0.008)	(0.011)	(0.008)	(0.011)	(0.008)	(0.009)	(0.006)	(0.005)	(0.006)	(0.006)	(0.006)	(0.005)
Age	0.030***	0.030***	0.030***	0.033***	0.030***	0.031***	0.029***	0.026***	0.029***	0.028***	0.029***	0.027***	0.013***	0.008***	0.013***	0.009***	0.013***	0.007***
	(0.004)	(0.003)	(0.004)	(0.003)	(0.004)	(0.003)	(0.004)	(0.003)	(0.004)	(0.003)	(0.004)	(0.003)	(0.003)	(0.002)	(0.003)	(0.002)	(0.003)	(0.002)
Age squared	-0.000***	-0.000***	-0.000***	-0.000***	-0.000***	-0.000***	-0.000***	-0.000***	-0.000***	-0.000***	-0.000***	-0.000***	-0.000***	-0.000***	-0.000***	-0.000***	-0.000***	-0.000***
	(0.000)	(0.000)	(0.000)	(0.000)	(0.000)	(0.000)	(0.000)	(0.000)	(0.000)	(0.000)	(0.000)	(0.000)	(0.000)	(0.000)	(0.000)	(0.000)	(0.000)	(0.000)
Urban	0.005	0.045**	0.013	0.055**	-0.082	-0.783***	-0.002	0.056***	-0.001	0.074***	-0.153***	-0.816***	0.011	0.029***	0.014	0.032*	-0.066	-0.980***
	(0.011)	(0.018)	(0.013)	(0.026)	(0.064)	(0.029)	(0.012)	(0.018)	(0.014)	(0.026)	(0.058)	(0.030)	(0.009)	(0.011)	(0.010)	(0.017)	(0.094)	(0.029)
Married	0.005	0.012	0.006	0.013	0.000	0.018	0.017**	0.004	0.018**	0.002	0.014*	0.007	0.003	0.004	0.001	0.001	-0.001	0.006
	(0.007)	(0.010)	(0.007)	(0.011)	(0.007)	(0.011)	(0.008)	(0.010)	(0.008)	(0.010)	(0.008)	(0.010)	(0.006)	(0.007)	(0.006)	(0.007)	(0.006)	(0.007)
Secondary education	-0.017*	0.023**	-0.017*	0.021**	-0.014	0.017*	0.002	0.034***	0.005	0.031***	0.009	0.021**	0.002	0.023***	0.001	0.024***	0.002	0.014***
	(0.009)	(0.010)	(0.009)	(0.010)	(0.010)	(0.009)	(0.009)	(0.009)	(0.010)	(0.010)	(0.009)	(0.009)	(0.007)	(0.005)	(0.008)	(0.006)	(0.007)	(0.005)
Tertiary education	-0.031***	0.053***	-0.031**	0.053***	-0.026**	0.042***	-0.014	0.069***	-0.010	0.067***	-0.007	0.052***	0.015	0.042***	0.017	0.043***	0.019*	0.038***
	(0.012)	(0.016)	(0.013)	(0.016)	(0.012)	(0.014)	(0.012)	(0.013)	(0.013)	(0.014)	(0.011)	(0.011)	(0.011)	(0.010)	(0.011)	(0.010)	(0.010)	(0.009)
Wealth quintile 2	0.046	0.046***	0.061**	0.042***	0.029	0.055***	0.030	0.049***	0.038	0.043***	0.009	0.047***	0.014	0.014**	0.013	0.011*	0.008	0.012*
	(0.031)	(0.014)	(0.028)	(0.016)	(0.028)	(0.015)	(0.025)	(0.012)	(0.025)	(0.013)	(0.024)	(0.013)	(0.011)	(0.006)	(0.013)	(0.007)	(0.014)	(0.006)
Wealth quintile 3	0.080*	0.076***	0.098***	0.064***	0.060*	0.056***	0.078**	0.078***	0.088***	0.068***	0.049*	0.048***	0.032**	0.037***	0.031*	0.030***	0.016	0.021***
	(0.041)	(0.015)	(0.037)	(0.017)	(0.034)	(0.016)	(0.032)	(0.014)	(0.033)	(0.015)	(0.029)	(0.014)	(0.015)	(0.008)	(0.017)	(0.008)	(0.016)	(0.007)
Wealth quintile 4	0.073*	0.110***	0.088**	0.099***	0.058*	0.068***	0.066**	0.112***	0.074**	0.100***	0.038	0.058***	0.045***	0.055***	0.044***	0.047***	0.030*	0.028***
	(0.041)	(0.017)	(0.037)	(0.018)	(0.033)	(0.018)	(0.031)	(0.015)	(0.031)	(0.016)	(0.028)	(0.017)	(0.015)	(0.010)	(0.016)	(0.010)	(0.016)	(0.008)
Wealth quintile 5	0.065	0.118***	0.083**	0.106***	0.059*	0.081***	0.060*	0.119***	0.069**	0.106***	0.036	0.067***	0.036**	0.063***	0.034**	0.053***	0.022	0.031**
	(0.041)	(0.020)	(0.038)	(0.021)	(0.034)	(0.021)	(0.031)	(0.020)	(0.031)	(0.021)	(0.028)	(0.022)	(0.015)	(0.015)	(0.016)	(0.015)	(0.017)	(0.015)
Tobacco user	0.008	0.020**	0.005	0.022**	0.004	0.022**	0.003	0.018*	0.001	0.025**	0.000	0.017*	0.017***	0.023***	0.018***	0.025***	0.017***	0.018***
	(0.009)	(0.009)	(0.009)	(0.010)	(0.009)	(0.009)	(0.008)	(0.010)	(0.008)	(0.010)	(0.008)	(0.009)	(0.005)	(0.006)	(0.006)	(0.006)	(0.006)	(0.007)
Alcohol user	-0.041***	0.013	-0.042***	0.013	-0.033***	0.002	-0.054***	-0.005	-0.054***	-0.006	-0.046***	-0.012	-0.032***	-0.010	-0.033***	-0.010	-0.028***	-0.009
	(0.010)	(0.010)	(0.011)	(0.011)	(0.012)	(0.010)	(0.010)	(0.009)	(0.010)	(0.010)	(0.011)	(0.010)	(0.006)	(0.007)	(0.006)	(0.007)	(0.006)	(0.006)
Diabetes	0.273***	0.226***	0.265***	0.215***	0.261***	0.195***	0.279***	0.221***	0.275***	0.220***	0.272***	0.202***	0.092***	0.079***	0.080***	0.080***	0.077***	0.072***
	(0.014)	(0.013)	(0.014)	(0.013)	(0.015)	(0.012)	(0.013)	(0.012)	(0.014)	(0.012)	(0.014)	(0.011)	(0.010)	(0.008)	(0.010)	(0.009)	(0.010)	(0.008)
Depressed	0.070***	0.078***	0.075***	0.086***	0.076***	0.090***	0.052***	0.057***	0.055***	0.062***	0.059***	0.068***	0.043***	0.021**	0.044***	0.022**	0.047***	0.028***
	(0.007)	(0.011)	(0.007)	(0.012)	(0.008)	(0.011)	(0.008)	(0.011)	(0.008)	(0.012)	(0.008)	(0.011)	(0.006)	(0.008)	(0.007)	(0.009)	(0.006)	(0.009)
Pregnant	0.038	-0.042	0.065	-0.030	0.060	-0.019	0.062	-0.033	0.081*	-0.033	0.076*	-0.012	0.049	0.042	0.057	0.048	0.052	0.047
	(0.040)	(0.043)	(0.041)	(0.047)	(0.041)	(0.044)	(0.042)	(0.039)	(0.043)	(0.042)	(0.043)	(0.041)	(0.037)	(0.029)	(0.041)	(0.032)	(0.042)	(0.033)
Recently lost job	0.003	0.020	0.007	0.022	0.002	0.005	-0.010	-0.016	-0.007	-0.012	-0.007	-0.026	0.010	-0.015	0.010	-0.019	0.011	-0.028
	(0.017)	(0.023)	(0.017)	(0.024)	(0.017)	(0.025)	(0.014)	(0.022)	(0.015)	(0.023)	(0.015)	(0.023)	(0.012)	(0.014)	(0.013)	(0.016)	(0.013)	(0.018)
Obese	0.094***	0.109***	0.095***	0.108***	0.092***	0.102***	0.097***	0.096***	0.099***	0.094***	0.097***	0.089***	0.007	0.009	0.006	0.010	0.004	0.007
	(0.007)	(0.010)	(0.008)	(0.011)	(0.008)	(0.010)	(0.007)	(0.010)	(0.007)	(0.011)	(0.007)	(0.010)	(0.005)	(0.006)	(0.006)	(0.006)	(0.005)	(0.006)
year5	-0.113***	0.234***	-0.022	0.128	0.052	0.102	0.062	0.185***	0.106***	0.093	0.054	0.219***	-0.008	0.095***	0.069***	-0.071	0.066	-0.072
	(0.040)	(0.051)	(0.052)	(0.177)	(0.049)	(0.092)	(0.045)	(0.052)	(0.036)	(0.175)	(0.067)	(0.043)	(0.012)	(0.022)	(0.025)	(0.211)	(0.048)	(0.062)
year7	-0.072**	0.218***	0.018	0.127	-0.031	0.019	0.010	0.158***	0.036***	0.088	-0.018	0.145***	-0.050**	0.081***	0.023*	-0.076	-0.027	-0.079*
	(0.033)	(0.048)	(0.013)	(0.178)	(0.027)	(0.087)	(0.031)	(0.049)	(0.011)	(0.177)	(0.054)	(0.030)	(0.021)	(0.021)	(0.013)	(0.213)	(0.035)	(0.046)
year8	-0.073**	0.274***	0.012	0.192	-0.020	0.077	0.014	0.200***	0.034***	0.140	-0.000	0.202***	-0.058***	0.154***	0.014	0.003	-0.038	-0.010
	(0.031)	(0.053)	(0.013)	(0.179)	(0.024)	(0.082)	(0.030)	(0.053)	(0.011)	(0.177)	(0.053)	(0.033)	(0.020)	(0.026)	(0.013)	(0.213)	(0.034)	(0.045)
year9	-0.056*	0.254***	0.025	0.164	-0.005	0.015	0.009	0.165***	0.028**	0.098	0.002	0.157***	-0.042**	0.109***	0.026*	-0.044	-0.038	-0.041
	(0.030)	(0.050)	(0.017)	(0.180)	(0.019)	(0.079)	(0.027)	(0.051)	(0.014)	(0.178)	(0.051)	(0.038)	(0.018)	(0.025)	(0.016)	(0.214)	(0.031)	(0.038)
year10		0.329***	0.133**	0.273		0.022		0.181***	0.065	0.168		0.023***		0.137***	0.071**	0.009		0.019
		(0.051)	(0.053)	(0.186)		(0.030)		(0.055)	(0.060)	(0.185)		(0.008)		(0.026)	(0.034)	(0.217)		(0.022)
country3	-0.126*		-0.060				-0.185**		-0.051				-0.114***		-0.058**			
	(0.066)		(0.066)				(0.074)		(0.051)				(0.021)		(0.029)			
country7	0.144***		0.222***				0.070		0.215***				0.071**		0.122***			
	(0.054)		(0.028)				(0.063)		(0.031)				(0.028)		(0.025)			
country8	-0.079		-0.008				-0.183***		-0.049*				-0.053*		0.002			
	(0.054)		(0.028)				(0.064)		(0.030)				(0.027)		(0.024)			
country9	0.044		0.123***				-0.071		0.071*				0.053		0.106***			
	(0.057)		(0.030)				(0.068)		(0.039)				(0.035)		(0.030)			
country11	0.051		0.135***				-0.018		0.127***				0.089***		0.143***			
	(0.055)		(0.029)				(0.063)		(0.031)				(0.027)		(0.025)			
country12	0.032		0.109***				-0.050		0.093***				-0.001		0.053**			

	(0.055)		(0.028)				(0.064)		(0.030)				(0.028)		(0.023)			
country13	0.044		0.118***				-0.042		0.096**				-0.047		0.005			
	(0.064)		(0.039)				(0.071)		(0.043)				(0.034)		(0.031)			
country14	-0.039		0.047				-0.163**		-0.017				-0.044*		0.014			
	(0.054)		(0.030)				(0.064)		(0.035)				(0.026)		(0.029)			
country16	-0.024		0.052				-0.135**		0.006				0.012		0.070**			
	(0.056)		(0.033)				(0.065)		(0.036)				(0.031)		(0.029)			
Public health facility available			0.064**	0.041					0.078*	0.062**					0.033**	0.027		
			(0.028)	(0.026)					(0.043)	(0.025)					(0.013)	(0.018)		
Private health facility available			0.010	0.007					0.012	-0.019					-0.007	0.001		
			(0.014)	(0.026)					(0.014)	(0.025)					(0.013)	(0.013)		
Any antihypertensive drug available			-0.013	-0.069*					-0.019	-0.094**					-0.003	-0.044		
			(0.037)	(0.039)					(0.039)	(0.040)					(0.030)	(0.028)		
Number of antihypertensive drug available			-0.002	0.010*					-0.001	0.010*					0.001	0.006		
			(0.004)	(0.006)					(0.004)	(0.006)					(0.004)	(0.004)		
Electric street lighting present			0.041*	0.035					0.043	0.035					0.019	0.018		
			(0.024)	(0.024)					(0.028)	(0.022)					(0.020)	(0.015)		
Traffic lights present			-0.022	-0.020					-0.014	-0.016					0.002	-0.007		
			(0.013)	(0.026)					(0.016)	(0.028)					(0.011)	(0.020)		
Completeness of road paving			-0.014	-0.018					-0.006	-0.003					-0.009	-0.009		
			(0.019)	(0.016)					(0.019)	(0.016)					(0.015)	(0.012)		
year6	-0.091***	0.224***		0.123	-0.041	0.059	-0.026	0.178***		0.096	-0.048	0.188***	-0.074***	0.092***		-0.072	-0.043	-0.063
	(0.032)	(0.047)		(0.177)	(0.029)	(0.087)	(0.031)	(0.048)		(0.175)	(0.055)	(0.025)	(0.023)	(0.018)		(0.212)	(0.037)	(0.046)
year12	0.038						0.002						0.147**					
	(0.082)						(0.090)						(0.068)					
year13	-0.020	-0.050					-0.010	-0.060**					0.097***	-0.031*				
	(0.054)	(0.055)					(0.060)	(0.025)					(0.031)	(0.018)				
country5	-0.061						-0.136**						-0.053*					
	(0.057)						(0.068)						(0.030)					
year4		0.115				0.078		0.087				0.224*		0.170				0.068
		(0.188)				(0.150)		(0.185)				(0.126)		(0.217)				(0.189)
year11		0.312***		0.260				0.169***		0.158				0.122***		-0.004		
		(0.053)		(0.188)				(0.053)		(0.185)				(0.033)		(0.218)		
year14		0.116**						0.118***						-0.155***				
		(0.053)						(0.026)						(0.019)				
country2		0.010		0.047				-0.013		0.018				-0.056***		-0.033		
		(0.028)		(0.034)				(0.026)		(0.032)				(0.017)		(0.025)		
country4		0.067**		0.058*				0.090***		0.070**				0.025		0.020		
		(0.033)		(0.034)				(0.031)		(0.032)				(0.022)		(0.023)		
country6		0.094**		0.120***				-0.066**		-0.036				-0.022		-0.005		
		(0.040)		(0.034)				(0.026)		(0.037)				(0.017)		(0.026)		
country10		0.024		0.014				0.084***		0.068**				0.002		-0.006		
		(0.035)		(0.036)				(0.032)		(0.033)				(0.026)		(0.027)		
country15		-0.056						-0.072*						-0.107***				
		(0.042)						(0.039)						(0.026)				
country17		-0.012		-0.005				0.002		0.013				-0.008		-0.003		
		(0.031)		(0.036)				(0.033)		(0.038)				(0.025)		(0.028)		
country18		0.017						-0.036						0.032				
		(0.055)						(0.046)						(0.021)				
country21		0.393***						0.310***						0.193***				
		(0.068)						(0.050)						(0.024)				
year3				-0.133						-0.109						-0.177		
				(0.191)						(0.188)						(0.220)		
Constant	-0.677***	-1.087***	-0.913***	-1.074***	-0.648***	-0.403***	-0.757***	-0.980***	-0.997***	-0.980***	-0.686***	-0.441***	-0.300***	-0.321***	-0.435***	-0.183	-0.234*	0.439***
	(0.123)	(0.086)	(0.116)	(0.214)	(0.132)	(0.130)	(0.118)	(0.083)	(0.124)	(0.212)	(0.133)	(0.095)	(0.083)	(0.056)	(0.089)	(0.232)	(0.129)	(0.081)
Observations	23,884	26,949	22,372	23,784	22,372	23,784	23,884	26,949	22,372	23,784	22,372	23,784	23,884	26,949	22,372	23,784	22,372	23,784
R-squared	0.141	0.103	0.138	0.099	0.103	0.058	0.158	0.121	0.157	0.117	0.123	0.062	0.058	0.053	0.054	0.052	0.026	0.012
Number of epochid					211	204					211	204					211	204

Robust standard errors in parentheses
*** p<0.01, ** p<0.05, * p<0.1

Effect of trust in organisations on hypertension detection, treatment and control among ALL hypertensives

Hypertension detection

VARIABLES	OLS+individual controls HIC & UMIC	OLS+individual controls LMIC & LIC	OLS+individual+ community controls HIC & UMIC	OLS+individual+ community controls LMIC & LIC	OLS+individual+ community controls + community fixed effects HIC & UMIC	OLS+individual+ community controls + community fixed effects LMIC & LIC
Trust in organisations	0.007	0.054**	0.009	0.063***	0.011	0.039*
	(0.009)	(0.021)	(0.010)	(0.023)	(0.010)	(0.023)
Female	0.084***	0.093***	0.084***	0.091***	0.084***	0.078***
	(0.008)	(0.012)	(0.008)	(0.013)	(0.008)	(0.010)
Age	0.030***	0.031***	0.031***	0.033***	0.030***	0.031***
	(0.004)	(0.003)	(0.004)	(0.003)	(0.004)	(0.003)
Age squared	-0.000***	-0.000***	-0.000***	-0.000***	-0.000***	-0.000***
	(0.000)	(0.000)	(0.000)	(0.000)	(0.000)	(0.000)
Urban	0.005	0.044**	0.013	0.055**	-0.083	-0.788***
	(0.011)	(0.018)	(0.013)	(0.026)	(0.064)	(0.029)
Married	0.005	0.011	0.006	0.011	0.001	0.017
	(0.007)	(0.010)	(0.008)	(0.011)	(0.007)	(0.011)
Secondary education	-0.018**	0.023**	-0.018*	0.020**	-0.015	0.017*
	(0.009)	(0.010)	(0.009)	(0.010)	(0.010)	(0.009)
Tertiary education	-0.033***	0.053***	-0.032**	0.052***	-0.028**	0.041***
	(0.012)	(0.016)	(0.013)	(0.016)	(0.013)	(0.014)
Wealth quintile 2	0.045	0.045***	0.062**	0.041***	0.032	0.054***
	(0.032)	(0.014)	(0.029)	(0.016)	(0.029)	(0.015)
Wealth quintile 3	0.077*	0.077***	0.096**	0.063***	0.059*	0.056***
	(0.042)	(0.016)	(0.039)	(0.017)	(0.034)	(0.016)
Wealth quintile 4	0.071*	0.109***	0.087**	0.098***	0.057*	0.068***
	(0.042)	(0.017)	(0.039)	(0.018)	(0.034)	(0.018)
Wealth quintile 5	0.061	0.114***	0.080**	0.102***	0.057*	0.078***
	(0.042)	(0.020)	(0.039)	(0.021)	(0.034)	(0.021)
Tobacco user	0.007	0.020**	0.005	0.022**	0.004	0.021**
	(0.009)	(0.010)	(0.009)	(0.010)	(0.009)	(0.009)
Alcohol user	-0.041***	0.015	-0.042***	0.014	-0.032***	0.002
	(0.010)	(0.010)	(0.011)	(0.011)	(0.012)	(0.010)
Diabetes	0.272***	0.226***	0.265***	0.215***	0.260***	0.195***
	(0.014)	(0.013)	(0.015)	(0.013)	(0.015)	(0.012)
Depressed	0.071***	0.075***	0.076***	0.084***	0.077***	0.088***
	(0.008)	(0.011)	(0.008)	(0.012)	(0.008)	(0.011)
Pregnant	0.034	-0.045	0.063	-0.035	0.059	-0.022
	(0.041)	(0.044)	(0.041)	(0.048)	(0.042)	(0.044)
Recently lost job	0.001	0.020	0.004	0.020	-0.000	0.005
	(0.017)	(0.023)	(0.017)	(0.024)	(0.017)	(0.024)
Obese	0.094***	0.110***	0.095***	0.108***	0.092***	0.102***
	(0.007)	(0.010)	(0.008)	(0.011)	(0.008)	(0.010)
year5	-0.217**	0.007	-0.024	0.261***	0.057	0.103
	(0.088)	(476.914)	(0.051)	(0.046)	(0.056)	(0.092)
year7	-0.171**	-0.010	0.021	0.260***	-0.025	0.021
	(0.073)	(552.791)	(0.013)	(0.044)	(0.040)	(0.086)
year8	-0.172**	0.048	0.014	0.326***	-0.014	0.080
	(0.073)	(389.186)	(0.013)	(0.050)	(0.038)	(0.082)
year9	-0.155**	0.026	0.028*	0.297***	0.002	0.021
	(0.074)	(413.572)	(0.016)	(0.047)	(0.034)	(0.078)
year10	-0.091	0.106	0.136**	0.410***		0.025
	(0.079)	(.)	(0.062)	(0.053)		(0.031)
country5			0.067			
			(0.066)			
country7	0.204***		0.289***			
	(0.024)		(0.063)			
country8	-0.019		0.059			
	(0.026)		(0.064)			
country9	0.103***		0.188***			
	(0.029)		(0.065)			
country11	0.111***		0.202***			
	(0.025)		(0.064)			
country12	0.090***		0.174***			

Hypertension treatment

VARIABLES	OLS+individual controls HIC & UMIC	OLS+individual controls LMIC & LIC	OLS+individual+ community controls HIC & UMIC	OLS+individual+ community controls LMIC & LIC	OLS+individual+ community controls + community fixed effects HIC & UMIC	OLS+individual+ community controls + community fixed effects LMIC & LIC
Trust in organisations	-0.007	0.027	-0.007	0.035	-0.002	0.000
	(0.009)	(0.023)	(0.010)	(0.025)	(0.010)	(0.024)
Female	0.082***	0.094***	0.082***	0.096***	0.082***	0.079***
	(0.008)	(0.011)	(0.008)	(0.011)	(0.008)	(0.009)
Age	0.030***	0.026***	0.029***	0.028***	0.029***	0.027***
	(0.004)	(0.003)	(0.004)	(0.003)	(0.004)	(0.003)
Age squared	-0.000***	-0.000***	-0.000***	-0.000***	-0.000***	-0.000***
	(0.000)	(0.000)	(0.000)	(0.000)	(0.000)	(0.000)
Urban	-0.003	0.056***	-0.003	0.074***	-0.154***	-0.820***
	(0.012)	(0.018)	(0.014)	(0.026)	(0.059)	(0.030)
Married	0.016**	0.002	0.017**	0.000	0.014*	0.005
	(0.008)	(0.010)	(0.008)	(0.010)	(0.008)	(0.010)
Secondary education	0.002	0.034***	0.005	0.031***	0.008	0.021**
	(0.009)	(0.009)	(0.010)	(0.010)	(0.009)	(0.008)
Tertiary education	-0.014	0.070***	-0.009	0.067***	-0.007	0.052***
	(0.012)	(0.013)	(0.013)	(0.014)	(0.011)	(0.011)
Wealth quintile 2	0.031	0.049***	0.039	0.042***	0.013	0.046***
	(0.025)	(0.012)	(0.026)	(0.013)	(0.025)	(0.013)
Wealth quintile 3	0.077**	0.078***	0.088**	0.068***	0.052*	0.048***
	(0.033)	(0.014)	(0.034)	(0.015)	(0.030)	(0.014)
Wealth quintile 4	0.066**	0.111***	0.075**	0.100***	0.041	0.057***
	(0.032)	(0.015)	(0.033)	(0.016)	(0.029)	(0.017)
Wealth quintile 5	0.058*	0.116***	0.068**	0.102***	0.038	0.064***
	(0.032)	(0.019)	(0.033)	(0.021)	(0.030)	(0.022)
Tobacco user	0.002	0.018*	0.000	0.024**	-0.001	0.016*
	(0.008)	(0.010)	(0.008)	(0.010)	(0.008)	(0.009)
Alcohol user	-0.054***	-0.003	-0.055***	-0.006	-0.046***	-0.012
	(0.009)	(0.009)	(0.010)	(0.010)	(0.011)	(0.010)
Diabetes	0.279***	0.222***	0.275***	0.221***	0.271***	0.202***
	(0.014)	(0.012)	(0.015)	(0.012)	(0.014)	(0.011)
Depressed	0.054***	0.055***	0.056***	0.061***	0.060***	0.067***
	(0.008)	(0.011)	(0.008)	(0.012)	(0.008)	(0.011)
Pregnant	0.072*	-0.030	0.087**	-0.028	0.082*	-0.007
	(0.042)	(0.039)	(0.043)	(0.042)	(0.044)	(0.041)
Recently lost job	-0.010	-0.019	-0.008	-0.015	-0.008	-0.028
	(0.014)	(0.022)	(0.014)	(0.023)	(0.015)	(0.023)
Obese	0.097***	0.096***	0.099***	0.094***	0.096***	0.089***
	(0.007)	(0.010)	(0.007)	(0.011)	(0.007)	(0.010)
year5	-0.078	0.031	0.107***	0.203***	0.051	0.221***
	(0.083)	(.)	(0.034)	(0.048)	(0.070)	(0.043)
year7	-0.128*	0.002	0.037***	0.197***	-0.022	0.148***
	(0.077)	(.)	(0.011)	(0.046)	(0.058)	(0.031)
year8	-0.124	0.046	0.035***	0.249***	-0.005	0.204***
	(0.077)	(.)	(0.011)	(0.050)	(0.058)	(0.033)
year9	-0.129*	0.011	0.029**	0.208***	-0.002	0.155***
	(0.078)	(.)	(0.014)	(0.049)	(0.056)	(0.039)
year10	-0.128	0.030	0.070	0.280***		0.025***
	(0.082)	(.)	(0.061)	(0.060)		(0.008)
country5			0.050			
			(0.049)			
country7	0.206***		0.267***			
	(0.030)		(0.046)			
country8	-0.047		0.002			
	(0.030)		(0.045)			
country9	0.066*		0.123**			
	(0.039)		(0.052)			
country11	0.117***		0.178***			
	(0.030)		(0.046)			
country12	0.083***		0.142***			

Hypertension control

VARIABLES	OLS+individual controls HIC & UMIC	OLS+individual controls LMIC & LIC	OLS+individual+ community controls HIC & UMIC	OLS+individual+ community controls LMIC & LIC	OLS+individual+ community controls + community fixed effects HIC & UMIC	OLS+individual+ community controls + community fixed effects LMIC & LIC
Trust in organisations	0.002	0.022	0.004	0.018	0.008	0.011
	(0.007)	(0.017)	(0.007)	(0.018)	(0.007)	(0.018)
Female	0.072***	0.031***	0.072***	0.031***	0.072***	0.023***
	(0.006)	(0.006)	(0.006)	(0.006)	(0.006)	(0.005)
Age	0.013***	0.009***	0.013***	0.009***	0.013***	0.007***
	(0.003)	(0.002)	(0.003)	(0.002)	(0.003)	(0.002)
Age squared	-0.000***	-0.000***	-0.000***	-0.000***	-0.000***	-0.000***
	(0.000)	(0.000)	(0.000)	(0.000)	(0.000)	(0.000)
Urban	0.011	0.029***	0.015	0.032*	-0.068	-0.980***
	(0.009)	(0.011)	(0.011)	(0.017)	(0.094)	(0.029)
Married	0.003	0.004	0.000	0.001	-0.001	0.006
	(0.006)	(0.007)	(0.006)	(0.007)	(0.006)	(0.007)
Secondary education	0.003	0.024***	0.001	0.025***	0.003	0.015***
	(0.007)	(0.005)	(0.008)	(0.006)	(0.008)	(0.005)
Tertiary education	0.016	0.043***	0.017	0.044***	0.020*	0.039***
	(0.011)	(0.010)	(0.011)	(0.010)	(0.011)	(0.009)
Wealth quintile 2	0.017	0.014**	0.017	0.011*	0.013	0.011*
	(0.011)	(0.006)	(0.014)	(0.007)	(0.014)	(0.006)
Wealth quintile 3	0.034**	0.038***	0.033*	0.031***	0.020	0.021***
	(0.015)	(0.008)	(0.017)	(0.009)	(0.017)	(0.007)
Wealth quintile 4	0.047***	0.055***	0.047***	0.047***	0.034**	0.028***
	(0.015)	(0.010)	(0.017)	(0.010)	(0.016)	(0.008)
Wealth quintile 5	0.037**	0.061***	0.036**	0.052***	0.024	0.030**
	(0.016)	(0.015)	(0.017)	(0.015)	(0.017)	(0.015)
Tobacco user	0.016***	0.024***	0.018***	0.025***	0.017***	0.018***
	(0.005)	(0.006)	(0.006)	(0.006)	(0.006)	(0.007)
Alcohol user	-0.033***	-0.010	-0.033***	-0.010	-0.028***	-0.010
	(0.006)	(0.007)	(0.006)	(0.007)	(0.006)	(0.006)
Diabetes	0.091***	0.080***	0.079***	0.080***	0.075***	0.073***
	(0.010)	(0.008)	(0.010)	(0.009)	(0.010)	(0.008)
Depressed	0.044***	0.020**	0.045***	0.022**	0.048***	0.028***
	(0.006)	(0.008)	(0.007)	(0.009)	(0.006)	(0.009)
Pregnant	0.052	0.044	0.062	0.050	0.056	0.048
	(0.038)	(0.029)	(0.042)	(0.032)	(0.043)	(0.033)
Recently lost job	0.009	-0.017	0.009	-0.022	0.010	-0.030
	(0.012)	(0.014)	(0.013)	(0.015)	(0.013)	(0.018)
Obese	0.007	0.011*	0.005	0.011*	0.004	0.008
	(0.005)	(0.006)	(0.006)	(0.006)	(0.006)	(0.006)
year5	-0.212***	0.028	0.067***	0.106***	0.071	-0.072
	(0.070)	(.)	(0.026)	(0.024)	(0.052)	(0.062)
year7	-0.250***	0.013	0.024*	0.101***	-0.020	-0.078*
	(0.068)	(235.302)	(0.013)	(0.024)	(0.040)	(0.046)
year8	-0.259***	0.086	0.015	0.180***	-0.032	-0.010
	(0.068)	(.)	(0.013)	(0.030)	(0.039)	(0.046)
year9	-0.242***	0.040	0.027*	0.132***	-0.030	-0.042
	(0.069)	(193.515)	(0.016)	(0.029)	(0.037)	(0.038)
year10	-0.198***	0.070	0.065*	0.186***		0.019
	(0.071)	(.)	(0.038)	(0.034)		(0.022)
country5			0.058*			
			(0.030)			
country7	0.124***		0.181***			
	(0.020)		(0.029)			
country8	-0.000		0.060**			
	(0.019)		(0.027)			
country9	0.102***		0.160***			
	(0.028)		(0.035)			
country11	0.141***		0.200***			
	(0.019)		(0.028)			
country12	0.049**		0.109***			

	(0.025)		(0.062)				(0.030)		(0.044)				(0.019)		(0.026)			
country13	0.105**		0.186***				0.093**		0.148***				0.005		0.063*			
	(0.043)		(0.069)				(0.046)		(0.052)				(0.027)		(0.034)			
country14	0.019		0.112*				-0.028		0.033				0.006		0.069***			
	(0.025)		(0.057)				(0.030)		(0.041)				(0.020)		(0.023)			
country16	0.034		0.117*				0.001		0.057				0.063**		0.127***			
	(0.029)		(0.064)				(0.034)		(0.044)				(0.025)		(0.030)			
Public health facility available			0.065**	0.041					0.077*	0.063**					0.033**	0.027		
			(0.029)	(0.026)					(0.042)	(0.025)					(0.013)	(0.018)		
Private health facility available			0.009	0.008					0.012	-0.018					-0.008	0.001		
			(0.014)	(0.026)					(0.014)	(0.025)					(0.013)	(0.013)		
Any antihypertensive drug available			-0.017	-0.067*					-0.017	-0.092**					-0.003	-0.044		
			(0.037)	(0.039)					(0.041)	(0.040)					(0.028)	(0.027)		
Number of antihypertensive drug available			-0.002	0.010*					-0.001	0.009*					0.001	0.006		
			(0.004)	(0.006)					(0.004)	(0.006)					(0.004)	(0.004)		
Electric street lighting present			0.041*	0.036					0.040	0.036					0.019	0.019		
			(0.025)	(0.024)					(0.028)	(0.022)					(0.020)	(0.015)		
Traffic lights present			-0.022	-0.021					-0.013	-0.016					0.002	-0.007		
			(0.014)	(0.026)					(0.016)	(0.028)					(0.010)	(0.020)		
Completeness of road paving			-0.013	-0.018					-0.005	-0.004					-0.009	-0.009		
			(0.019)	(0.016)					(0.019)	(0.016)					(0.015)	(0.012)		
year6	-0.192***	-0.003		0.256***	-0.038	0.062	-0.165**	0.023		0.205***	-0.053	0.191***	-0.276***	0.024		0.105***	-0.037	-0.062
	(0.074)	(423.000)		(0.040)	(0.041)	(0.087)	(0.077)	(.)		(0.043)	(0.059)	(0.026)	(0.069)	(.)		(0.020)	(0.042)	(0.046)
year13	-0.058	-0.059					-0.012	-0.060**					-0.052	-0.038*				
	(0.077)	(0.055)					(0.075)	(0.025)					(0.072)	(0.020)				
year14	-0.039	0.085					-0.004	0.096***					-0.149**	-0.173***				
	(0.082)	(0.060)					(0.090)	(0.030)					(0.068)	(0.022)				
country3	-0.072						-0.050						-0.061**					
	(0.063)						(0.048)						(0.026)					
year3		-0.228						-0.154						-0.067				
		(154.838)						(.)						(282.076)				
year4		-0.112		0.133		0.083		-0.067		0.109		0.231*		0.102		0.177		0.069
		(768.727)		(0.190)		(0.149)		(.)		(0.188)		(0.125)		(.)		(0.220)		(0.189)
year11		0.086		0.394***				0.016		0.268***				0.054		0.172***		
		(11.932)		(0.054)				(.)		(0.057)				(.)		(0.037)		
country2		0.012		0.049				-0.011		0.019				-0.055***		-0.033		
		(0.028)		(0.035)				(0.027)		(0.033)				(0.017)		(0.025)		
country4		0.061*		0.051				0.087***		0.066**				0.024		0.020		
		(0.033)		(0.034)				(0.031)		(0.033)				(0.022)		(0.024)		
country6		0.084**		0.109***				-0.073***		-0.044				-0.025		-0.008		
		(0.041)		(0.035)				(0.027)		(0.036)				(0.017)		(0.025)		
country10		0.026		0.014				0.085***		0.069**				0.003		-0.005		
		(0.035)		(0.037)				(0.032)		(0.034)				(0.026)		(0.027)		
country15		-0.050						-0.069*						-0.106***				
		(0.043)						(0.039)						(0.026)				
country17		-0.012		-0.005				0.002		0.012				-0.007		-0.002		
		(0.031)		(0.036)				(0.033)		(0.038)				(0.025)		(0.028)		
country18		-0.200						-0.188						-0.029				
		(517.628)						(.)						(.)				
country21		0.166						0.144						0.127				
		(395.454)						(.)						(.)				
Constant	-0.644***	-0.867	-0.989***	-1.210***	-0.664***	-0.406***	-0.759***	-0.829	-1.052***	-1.091***	-0.689***	-0.442***	-0.146	-0.258	-0.496***	-0.363***	-0.244*	0.434***
	(0.132)	(471.484)	(0.112)	(0.106)	(0.133)	(0.131)	(0.122)	(.)	(0.123)	(0.108)	(0.135)	(0.095)	(0.103)	(.)	(0.085)	(0.077)	(0.131)	(0.082)
Observations	23,682	26,809	22,222	23,756	22,222	23,756	23,682	26,809	22,222	23,756	22,222	23,756	23,682	26,809	22,222	23,756	22,222	23,756
R-squared	0.142	0.103	0.139	0.099	0.104	0.058	0.159	0.120	0.157	0.117	0.124	0.062	0.058	0.053	0.054	0.052	0.026	0.012
Number of epochid					211	204					211	204					211	204

Robust standard errors in parentheses
*** p<0.01, ** p<0.05, * p<0.1

Effect of membership of any social organisation on hypertension treatment and control among KNOWN hypertensives only

	Hypertension treatment						Hypertension control					
	OLS+individual controls		OLS+individual+ community controls		OLS+individual+ community controls + community fixed effects		OLS+individual controls		OLS+individual+ community controls		OLS+individual+ community controls + community fixed effects	
VARIABLES	HIC & UMIC	LMIC & LIC	HIC & UMIC	LMIC & LIC	HIC & UMIC	LMIC & LIC	HIC & UMIC	LMIC & LIC	HIC & UMIC	LMIC & LIC	HIC & UMIC	LMIC & LIC
Membership	0.003	0.007	-0.000	0.007	-0.002	0.014	-0.011	0.035**	-0.009	0.039**	-0.001	0.032*
	(0.007)	(0.012)	(0.007)	(0.012)	(0.007)	(0.012)	(0.009)	(0.015)	(0.009)	(0.016)	(0.009)	(0.016)
Female	0.022***	0.057***	0.021**	0.066***	0.025***	0.057***	0.094***	0.047***	0.092***	0.051***	0.096***	0.041***
	(0.008)	(0.016)	(0.009)	(0.017)	(0.008)	(0.015)	(0.009)	(0.014)	(0.009)	(0.015)	(0.009)	(0.014)
Age	0.024***	0.030***	0.024***	0.022***	0.024***	0.019***	0.005	0.004	0.005	0.003	0.006	-0.000
	(0.005)	(0.007)	(0.005)	(0.007)	(0.005)	(0.007)	(0.006)	(0.008)	(0.006)	(0.008)	(0.007)	(0.008)
Age squared	-0.000***	-0.000***	-0.000***	-0.000**	-0.000***	-0.000**	-0.000	-0.000	-0.000	-0.000	-0.000	-0.000
	(0.000)	(0.000)	(0.000)	(0.000)	(0.000)	(0.000)	(0.000)	(0.000)	(0.000)	(0.000)	(0.000)	(0.000)
Urban	-0.010	0.025	-0.010	0.027	-0.118***	0.387***	0.015	0.021	0.020	0.031	-0.023	-0.033
	(0.012)	(0.023)	(0.016)	(0.027)	(0.020)	(0.066)	(0.015)	(0.024)	(0.018)	(0.027)	(0.119)	(0.089)
Married	0.011	-0.024*	0.011	-0.021	0.012	-0.009	0.004	-0.004	-0.002	-0.005	0.000	0.011
	(0.007)	(0.012)	(0.008)	(0.013)	(0.008)	(0.013)	(0.010)	(0.016)	(0.010)	(0.017)	(0.011)	(0.018)
Secondary education	0.024***	0.056***	0.029***	0.047***	0.028***	0.024*	0.014	0.052***	0.011	0.050***	0.009	0.022
	(0.008)	(0.018)	(0.009)	(0.016)	(0.008)	(0.014)	(0.012)	(0.018)	(0.012)	(0.019)	(0.012)	(0.020)
Tertiary education	0.020*	0.090***	0.027**	0.085***	0.024***	0.064***	0.055***	0.108***	0.058***	0.112***	0.055***	0.085***
	(0.011)	(0.019)	(0.012)	(0.019)	(0.009)	(0.017)	(0.017)	(0.027)	(0.018)	(0.028)	(0.018)	(0.028)
Wealth quintile 2	0.033	0.027	0.029	0.004	0.011	0.005	0.040	0.027	0.031	0.015	0.021	0.006
	(0.029)	(0.018)	(0.032)	(0.021)	(0.033)	(0.018)	(0.027)	(0.020)	(0.032)	(0.021)	(0.034)	(0.021)
Wealth quintile 3	0.085***	0.014	0.082**	-0.013	0.052	-0.001	0.058*	0.040	0.049	0.024	0.019	0.017
	(0.029)	(0.023)	(0.033)	(0.026)	(0.034)	(0.023)	(0.031)	(0.025)	(0.037)	(0.025)	(0.038)	(0.026)
Wealth quintile 4	0.084***	0.042*	0.081**	0.012	0.045	0.022	0.086***	0.057*	0.079**	0.027	0.045	0.019
	(0.028)	(0.025)	(0.032)	(0.030)	(0.033)	(0.024)	(0.030)	(0.032)	(0.034)	(0.029)	(0.036)	(0.028)
Wealth quintile 5	0.085***	0.034	0.080**	0.010	0.044	0.020	0.074**	0.058	0.063*	0.028	0.029	0.014
	(0.029)	(0.031)	(0.032)	(0.036)	(0.034)	(0.030)	(0.032)	(0.036)	(0.036)	(0.034)	(0.038)	(0.035)
Tobacco user	-0.007	0.016	-0.008	0.021	-0.008	0.020	0.022**	0.046**	0.027***	0.051***	0.027***	0.044**
	(0.007)	(0.012)	(0.007)	(0.013)	(0.007)	(0.013)	(0.009)	(0.018)	(0.009)	(0.019)	(0.009)	(0.020)
Alcohol user	-0.027***	-0.021	-0.027***	-0.025	-0.019**	-0.034**	-0.032***	-0.020	-0.030***	-0.016	-0.023**	-0.024
	(0.009)	(0.019)	(0.009)	(0.021)	(0.009)	(0.016)	(0.010)	(0.021)	(0.010)	(0.023)	(0.010)	(0.021)
Diabetes	0.056***	0.045***	0.059***	0.042***	0.057***	0.035***	0.008	0.016	-0.002	0.013	-0.007	0.016
	(0.007)	(0.011)	(0.007)	(0.011)	(0.007)	(0.011)	(0.011)	(0.015)	(0.011)	(0.016)	(0.011)	(0.017)
Depressed	-0.005	-0.014	-0.007	-0.011	-0.001	-0.000	0.033***	0.003	0.032***	-0.001	0.038***	0.001
	(0.006)	(0.012)	(0.006)	(0.011)	(0.006)	(0.011)	(0.009)	(0.017)	(0.010)	(0.018)	(0.009)	(0.018)
Pregnant	0.071*	0.067	0.055	0.034	0.043	0.054	0.063	0.139	0.056	0.111	0.029	0.064
	(0.040)	(0.057)	(0.041)	(0.058)	(0.041)	(0.055)	(0.057)	(0.101)	(0.059)	(0.101)	(0.062)	(0.106)
Recently lost job	-0.007	-0.042	-0.009	-0.055	-0.002	-0.053	0.021	-0.063**	0.019	-0.078***	0.020	-0.042
	(0.018)	(0.038)	(0.018)	(0.039)	(0.018)	(0.037)	(0.021)	(0.026)	(0.021)	(0.027)	(0.021)	(0.029)
Obese	0.021***	0.006	0.020***	0.005	0.019***	0.007	-0.039***	-0.037***	-0.042***	-0.037**	-0.041***	-0.036**
	(0.007)	(0.011)	(0.007)	(0.011)	(0.007)	(0.011)	(0.009)	(0.014)	(0.009)	(0.014)	(0.009)	(0.014)
year6	-0.094***	0.051	-0.123***	0.033	-0.056	0.162	-0.144***	-0.144***	-0.128**	-0.131**	-0.080	0.071

	(0.030)	(0.058)	(0.037)	(0.061)	(0.059)	(0.116)	(0.045)	(0.052)	(0.056)	(0.052)	(0.051)	(0.102)
year7	-0.052	0.007	-0.085**	-0.009	-0.018	0.144	-0.111***	-0.184***	-0.099*	-0.193***	-0.061	0.087
	(0.032)	(0.060)	(0.034)	(0.063)	(0.058)	(0.121)	(0.042)	(0.056)	(0.053)	(0.054)	(0.046)	(0.107)
year8	-0.041	0.037	-0.074**	0.016	-0.004	0.197	-0.130***	-0.018	-0.117**	-0.009	-0.093**	0.205*
	(0.029)	(0.062)	(0.033)	(0.065)	(0.057)	(0.123)	(0.041)	(0.061)	(0.052)	(0.060)	(0.044)	(0.116)
year9	-0.069**	-0.013	-0.101***	-0.032	-0.018	0.010	-0.114**	-0.079	-0.110**	-0.074	-0.105***	0.106
	(0.032)	(0.064)	(0.033)	(0.070)	(0.056)	(0.253)	(0.044)	(0.066)	(0.052)	(0.066)	(0.039)	(0.155)
year10	-0.050	0.009	-0.111*	-0.019		0.001	-0.028	-0.081	-0.048	-0.259**		0.038***
	(0.043)	(0.072)	(0.058)	(0.153)		(0.005)	(0.057)	(0.068)	(0.040)	(0.100)		(0.007)
country3	-0.117***		-0.106***				-0.097*		-0.084			
	(0.032)		(0.036)				(0.050)		(0.054)			
country7	0.039**		0.046*				0.108***		0.100***			
	(0.019)		(0.026)				(0.027)		(0.037)			
country8	-0.090***		-0.103***				-0.005		-0.005			
	(0.025)		(0.029)				(0.029)		(0.037)			
country9	-0.055*		-0.059*				0.130***		0.123***			
	(0.030)		(0.034)				(0.036)		(0.041)			
country11	-0.008		-0.014				0.198***		0.193***			
	(0.018)		(0.026)				(0.028)		(0.038)			
country12	-0.002		0.001				0.037		0.035			
	(0.019)		(0.024)				(0.027)		(0.033)			
country13	-0.035*		-0.040				-0.048		-0.054			
	(0.020)		(0.026)				(0.038)		(0.046)			
country14	-0.113***		-0.100***				0.005		0.011			
	(0.025)		(0.035)				(0.033)		(0.046)			
country16	-0.069***		-0.070**				0.097***		0.105**			
	(0.021)		(0.027)				(0.035)		(0.042)			
Public health facility available			-0.000	0.035					0.057*	-0.093		
			(0.027)	(0.136)					(0.033)	(0.070)		
Private health facility available			-0.011	0.037					-0.019	0.003		
			(0.018)	(0.045)					(0.021)	(0.041)		
Any antihypertensive drug available			0.034	0.134					0.009	0.097*		
			(0.065)	(0.096)					(0.052)	(0.059)		
Number of antihypertensive drug available			0.004	-0.012					0.003	-0.030***		
			(0.006)	(0.008)					(0.008)	(0.008)		
Electric street lighting present			0.041	0.131*					0.031	0.097***		
			(0.038)	(0.070)					(0.034)	(0.027)		
Traffic lights present			-0.003	-0.014					0.008	0.039		
			(0.021)	(0.025)					(0.019)	(0.024)		
Completeness of road paving			0.004	0.021					-0.014	-0.020		
			(0.021)	(0.021)					(0.023)	(0.019)		
year5	-0.007	-0.027		-0.052	-0.018	-0.001	-0.026	-0.193***		-0.194***	0.076	-0.054
	(0.044)	(0.063)		(0.062)	(0.073)	(0.111)	(0.060)	(0.055)		(0.055)	(0.083)	(0.104)
year12	-0.072						0.211**					
	(0.074)						(0.092)					
year13	-0.032	-0.130					0.163***	-0.112				
	(0.032)	(0.175)					(0.048)	(0.082)				
year2		0.052		0.048		0.086*		-0.029		-0.014		0.001

		(0.058)		(0.058)		(0.044)		(0.054)		(0.054)		(0.042)
year3		-0.068		-0.095		-0.082		-0.158**		-0.157**		-0.055
		(0.129)		(0.124)		(0.057)		(0.073)		(0.075)		(0.090)
year4		-0.031		-0.046		0.037		-0.115*		-0.100*		0.019
		(0.067)		(0.065)		(0.091)		(0.058)		(0.059)		(0.095)
year11		0.016		-0.015				-0.111		-0.291***		
		(0.074)		(0.154)				(0.069)		(0.100)		
year14		-0.041						-0.348***				
		(0.184)						(0.085)				
country4		0.059***		0.036*				-0.007		-0.013		
		(0.022)		(0.021)				(0.031)		(0.034)		
country6		-0.302***		-0.279***				-0.126***		-0.116**		
		(0.024)		(0.064)				(0.036)		(0.048)		
country10		0.052**		0.042*				-0.049		-0.031		
		(0.026)		(0.023)				(0.040)		(0.038)		
country15		-0.206***						-0.277***				
		(0.045)						(0.049)				
country17		-0.044		-0.054				-0.046		-0.082		
		(0.046)		(0.040)				(0.050)		(0.054)		
country18		-0.519***						-0.285***				
		(0.133)						(0.095)				
country21		0.119						-0.002				
		(0.186)						(0.098)				
Constant	0.024	-0.212	-0.027	-0.267	0.059	-0.223	0.155	0.327	0.094	0.480*	0.227	0.308
	(0.143)	(0.203)	(0.187)	(0.285)	(0.176)	(0.210)	(0.180)	(0.210)	(0.204)	(0.247)	(0.234)	(0.242)
Observations	12,403	5,387	11,683	4,912	11,683	4,912	12,403	5,387	11,683	4,912	11,683	4,912
R-squared	0.071	0.106	0.071	0.091	0.040	0.042	0.051	0.046	0.050	0.045	0.019	0.015
Number of epochid					209	160					209	160

Robust standard errors in parentheses
*** p<0.01, ** p<0.05, * p<0.1

Effect of trust in people on hypertension treatment and control among KNOWN hypertensives only

	Hypertension treatment						Hypertension control					
	OLS+individual controls		OLS+individual+ community controls		OLS+individual+ community controls + community fixed effects		OLS+individual controls		OLS+individual+ community controls		OLS+individual+ community controls + community fixed effects	
VARIABLES	HIC & UMIC	LMIC & LIC	HIC & UMIC	LMIC & LIC	HIC & UMIC	LMIC & LIC	HIC & UMIC	LMIC & LIC	HIC & UMIC	LMIC & LIC	HIC & UMIC	LMIC & LIC
Trust in people	0.006	-0.012	0.001	-0.009	0.005	-0.007	0.011	0.001	0.009	0.003	0.007	0.004
	(0.007)	(0.009)	(0.007)	(0.009)	(0.007)	(0.009)	(0.010)	(0.010)	(0.011)	(0.010)	(0.010)	(0.008)
Female	0.022***	0.046***	0.021**	0.051***	0.025***	0.038***	0.093***	0.019*	0.092***	0.018	0.096***	0.008
	(0.008)	(0.010)	(0.009)	(0.010)	(0.008)	(0.010)	(0.009)	(0.011)	(0.009)	(0.011)	(0.009)	(0.010)
Age	0.024***	0.016***	0.023***	0.013***	0.024***	0.013***	0.005	0.002	0.005	0.001	0.006	-0.001
	(0.005)	(0.005)	(0.005)	(0.005)	(0.005)	(0.005)	(0.006)	(0.005)	(0.006)	(0.006)	(0.007)	(0.005)
Age squared	-0.000***	-0.000**	-0.000***	-0.000*	-0.000***	-0.000*	-0.000	-0.000	-0.000	-0.000	-0.000	-0.000
	(0.000)	(0.000)	(0.000)	(0.000)	(0.000)	(0.000)	(0.000)	(0.000)	(0.000)	(0.000)	(0.000)	(0.000)
Urban	-0.011	0.053***	-0.010	0.070***	-0.116***	-0.195***	0.015	0.054***	0.020	0.052*	-0.020	-0.815***
	(0.012)	(0.015)	(0.016)	(0.022)	(0.020)	(0.021)	(0.015)	(0.019)	(0.018)	(0.027)	(0.119)	(0.042)
Married	0.012	-0.017	0.011	-0.018	0.012	-0.020*	0.004	-0.000	-0.001	-0.005	0.001	-0.001
	(0.007)	(0.011)	(0.008)	(0.011)	(0.008)	(0.011)	(0.010)	(0.013)	(0.010)	(0.014)	(0.011)	(0.014)
Secondary education	0.024***	0.031***	0.029***	0.028***	0.028***	0.012	0.014	0.033***	0.010	0.034***	0.009	0.016*
	(0.008)	(0.010)	(0.009)	(0.011)	(0.008)	(0.009)	(0.012)	(0.010)	(0.012)	(0.011)	(0.012)	(0.009)
Tertiary education	0.020*	0.057***	0.027**	0.054***	0.024**	0.042***	0.054***	0.058***	0.056***	0.058***	0.055***	0.055***
	(0.011)	(0.013)	(0.012)	(0.014)	(0.009)	(0.013)	(0.017)	(0.018)	(0.018)	(0.019)	(0.018)	(0.017)
Wealth quintile 2	0.031	0.042**	0.030	0.030	0.012	0.010	0.033	0.015	0.024	0.005	0.014	0.006
	(0.029)	(0.021)	(0.033)	(0.021)	(0.034)	(0.016)	(0.028)	(0.014)	(0.034)	(0.016)	(0.035)	(0.016)
Wealth quintile 3	0.085***	0.055**	0.083**	0.047**	0.053	0.010	0.052	0.055***	0.043	0.039**	0.012	0.025
	(0.030)	(0.023)	(0.034)	(0.023)	(0.034)	(0.018)	(0.032)	(0.017)	(0.038)	(0.018)	(0.040)	(0.017)
Wealth quintile 4	0.084***	0.064***	0.082**	0.054**	0.046	0.013	0.079***	0.072***	0.071**	0.054***	0.038	0.035*
	(0.028)	(0.022)	(0.032)	(0.022)	(0.033)	(0.019)	(0.030)	(0.019)	(0.036)	(0.019)	(0.038)	(0.019)
Wealth quintile 5	0.086***	0.051*	0.082**	0.039	0.045	0.006	0.066**	0.070***	0.056	0.052**	0.022	0.033
	(0.029)	(0.027)	(0.033)	(0.027)	(0.034)	(0.026)	(0.032)	(0.025)	(0.037)	(0.025)	(0.040)	(0.024)
Tobacco user	-0.007	0.006	-0.008	0.016	-0.008	-0.001	0.023**	0.043***	0.028***	0.044***	0.028***	0.035***
	(0.007)	(0.012)	(0.007)	(0.012)	(0.007)	(0.012)	(0.009)	(0.012)	(0.009)	(0.013)	(0.009)	(0.013)
Alcohol user	-0.027***	-0.038***	-0.026***	-0.042***	-0.018**	-0.032***	-0.032***	-0.032**	-0.029***	-0.033**	-0.023**	-0.027**
	(0.009)	(0.012)	(0.009)	(0.012)	(0.009)	(0.011)	(0.010)	(0.014)	(0.010)	(0.014)	(0.010)	(0.013)
Diabetes	0.056***	0.043***	0.059***	0.048***	0.057***	0.045***	0.009	0.035***	-0.001	0.037***	-0.007	0.031***
	(0.007)	(0.008)	(0.007)	(0.008)	(0.007)	(0.008)	(0.011)	(0.011)	(0.011)	(0.012)	(0.011)	(0.012)
Depressed	-0.005	-0.020*	-0.007	-0.020*	-0.001	-0.012	0.033***	-0.002	0.032***	-0.004	0.039***	0.007
	(0.006)	(0.012)	(0.006)	(0.012)	(0.006)	(0.012)	(0.009)	(0.015)	(0.010)	(0.016)	(0.009)	(0.016)
Pregnant	0.071*	-0.004	0.055	-0.015	0.044	0.023	0.064	0.105	0.057	0.105	0.030	0.097
	(0.040)	(0.062)	(0.041)	(0.058)	(0.041)	(0.051)	(0.057)	(0.065)	(0.059)	(0.066)	(0.062)	(0.072)
Recently lost job	-0.008	-0.077***	-0.009	-0.072**	-0.002	-0.067**	0.022	-0.027	0.019	-0.037	0.020	-0.039
	(0.018)	(0.028)	(0.018)	(0.029)	(0.018)	(0.027)	(0.021)	(0.026)	(0.021)	(0.029)	(0.021)	(0.033)
Obese	0.021***	0.013	0.020***	0.011	0.019***	0.013	-0.038***	-0.030***	-0.042***	-0.028***	-0.040***	-0.026**
	(0.007)	(0.009)	(0.007)	(0.010)	(0.007)	(0.009)	(0.009)	(0.010)	(0.009)	(0.011)	(0.009)	(0.010)
year6	-0.095***	-0.018	-0.124***	-0.082***	-0.059	0.471***	-0.144***	-0.023	-0.128**	-0.657***	-0.081	-0.135***

	(0.030)	(1,183.024)	(0.037)	(0.029)	(0.057)	(0.016)	(0.045)	(2,082.193)	(0.055)	(0.037)	(0.052)	(0.018)
year7	-0.054*	-0.046	-0.086**	-0.101***	-0.022	0.446***	-0.111***	-0.037	-0.100*	-0.660***	-0.061	-0.148***
	(0.032)	(1,021.090)	(0.035)	(0.028)	(0.055)	(0.021)	(0.042)	(1,527.095)	(0.052)	(0.038)	(0.047)	(0.032)
year8	-0.042	-0.058	-0.075**	-0.107***	-0.007	0.482***	-0.129***	0.076	-0.117**	-0.542***	-0.093**	-0.031
	(0.029)	(851.130)	(0.034)	(0.034)	(0.055)	(0.025)	(0.041)	(1,940.557)	(0.051)	(0.047)	(0.045)	(0.041)
year9	-0.070**	-0.095	-0.102***	-0.146***	-0.021	0.444***	-0.114**	0.008	-0.110**	-0.607***	-0.107***	-0.070
	(0.032)	(1,574.127)	(0.033)	(0.035)	(0.054)	(0.028)	(0.044)	(2,073.566)	(0.050)	(0.047)	(0.040)	(0.076)
year10	-0.049	-0.149	-0.110*	-0.132		-0.008	-0.025	0.007	-0.042	-0.567***		0.021
	(0.043)	(1,191.141)	(0.058)	(0.096)		(0.018)	(0.057)	(2,103.900)	(0.039)	(0.053)		(0.020)
country5			0.109***						0.096*			
			(0.037)						(0.053)			
country7	0.040**		0.155***				0.103***		0.192***			
	(0.018)		(0.046)				(0.027)		(0.056)			
country8	-0.090***		0.005				-0.012		0.087			
	(0.025)		(0.040)				(0.029)		(0.053)			
country9	-0.053*		0.049				0.128***		0.219***			
	(0.029)		(0.046)				(0.035)		(0.058)			
country11	-0.006		0.095**				0.193***		0.286***			
	(0.018)		(0.043)				(0.027)		(0.054)			
country12	0.001		0.110***				0.039		0.133***			
	(0.019)		(0.038)				(0.027)		(0.051)			
country13	-0.034*		0.068				-0.049		0.042			
	(0.020)		(0.043)				(0.039)		(0.061)			
country14	-0.109***		0.009				0.004		0.106**			
	(0.025)		(0.042)				(0.033)		(0.051)			
country16	-0.068***		0.038				0.096***		0.201***			
	(0.021)		(0.039)				(0.035)		(0.056)			
Public health facility available			0.000	0.081***					0.058*	0.048		
			(0.027)	(0.030)					(0.033)	(0.033)		
Private health facility available			-0.010	-0.039					-0.019	0.012		
			(0.017)	(0.025)					(0.021)	(0.025)		
Any antihypertensive drug available			0.033	-0.097***					0.008	-0.077		
			(0.065)	(0.033)					(0.052)	(0.048)		
Number of antihypertensive drug available			0.004	0.007					0.003	0.010		
			(0.006)	(0.005)					(0.008)	(0.008)		
Electric street lighting present			0.042	0.040*					0.028	0.033		
			(0.039)	(0.021)					(0.034)	(0.029)		
Traffic lights present			-0.003	-0.015					0.008	-0.007		
			(0.021)	(0.021)					(0.020)	(0.030)		
Completeness of road paving			0.004	0.015					-0.014	-0.016		
			(0.021)	(0.015)					(0.023)	(0.019)		
year5	-0.005	-0.016		-0.093***	-0.020	0.478***	-0.024	-0.024		-0.655***	0.075	-0.160**
	(0.044)	(1,281.369)		(0.032)	(0.071)	(0.031)	(0.060)	(1,977.001)		(0.042)	(0.083)	(0.062)
year12	-0.074	-0.561					0.209**	-0.130				
	(0.074)	(1,240.429)					(0.093)	(2,182.998)				
year13	-0.033	-0.715					0.163***	-0.271				
	(0.032)	(1,635.235)					(0.048)	(2,097.121)				
country3	-0.115***						-0.110**					

	(0.032)						(0.050)					
year4		0.032				0.537***		0.624				0.481***
		(1,444.948)				(0.030)		(1,998.893)				(0.046)
year11		-0.144		-0.130				-0.009		-0.585***		
		(1,265.866)		(0.088)				(1,868.602)		(0.057)		
year14		-0.617						-0.507				
		(374.024)						(2,161.679)				
country2		-0.045***		-0.022				-0.134***		-0.096**		
		(0.016)		(0.025)				(0.028)		(0.043)		
country4		0.064***		0.039				0.007		0.005		
		(0.023)		(0.024)				(0.034)		(0.036)		
country6		-0.276***		-0.253***				-0.090**		-0.065		
		(0.026)		(0.047)				(0.037)		(0.052)		
country10		0.060***		0.043*				-0.021		-0.032		
		(0.023)		(0.025)				(0.040)		(0.042)		
country15		-0.162***						-0.227***				
		(0.047)						(0.049)				
country17		-0.001		0.014				-0.020		-0.006		
		(0.047)		(0.056)				(0.040)		(0.045)		
country21		0.632***						0.282***				
		(0.127)						(0.023)				
Constant	0.022	0.186	-0.134	0.278*	0.058	-0.001	0.155	0.205	0.004	0.865***	0.228	0.937***
	(0.143)	(1,181.332)	(0.190)	(0.154)	(0.175)	(0.141)	(0.180)	(2,333.436)	(0.191)	(0.167)	(0.234)	(0.156)
Observations	12,412	11,589	11,679	10,516	11,679	10,516	12,412	11,589	11,679	10,516	11,679	10,516
R-squared	0.070	0.071	0.071	0.067	0.040	0.019	0.051	0.054	0.050	0.053	0.019	0.009
Number of epochid					209	202					209	202

Robust standard errors in parentheses
*** p<0.01, ** p<0.05, * p<0.1

Effect of trust in organisations on hypertension treatment and control among KNOWN hypertensives only

	Hypertension treatment						Hypertension control					
	OLS+individual controls		OLS+individual+ community controls		OLS+individual+ community controls + community fixed effects		OLS+individual controls		OLS+individual+ community controls		OLS+individual+ community controls + community fixed effects	
VARIABLES	HIC & UMIC	LMIC & LIC	HIC & UMIC	LMIC & LIC	HIC & UMIC	LMIC & LIC	HIC & UMIC	LMIC & LIC	HIC & UMIC	LMIC & LIC	HIC & UMIC	LMIC & LIC
Trust in organisations	-0.013	-0.036	-0.017*	-0.025	-0.014	-0.037	-0.003	0.007	-0.001	0.002	0.008	-0.002
	(0.010)	(0.028)	(0.010)	(0.029)	(0.009)	(0.029)	(0.011)	(0.026)	(0.012)	(0.027)	(0.012)	(0.029)
Female	0.022***	0.047***	0.020**	0.051***	0.025***	0.038***	0.093***	0.020*	0.092***	0.019*	0.095***	0.009
	(0.008)	(0.010)	(0.009)	(0.010)	(0.008)	(0.010)	(0.009)	(0.011)	(0.009)	(0.011)	(0.009)	(0.010)
Age	0.024***	0.016***	0.024***	0.013***	0.024***	0.013***	0.004	0.002	0.005	0.001	0.006	-0.001
	(0.005)	(0.005)	(0.005)	(0.005)	(0.005)	(0.005)	(0.006)	(0.005)	(0.006)	(0.006)	(0.007)	(0.005)
Age squared	-0.000***	-0.000**	-0.000***	-0.000*	-0.000***	-0.000*	-0.000	-0.000	-0.000	-0.000	-0.000	-0.000
	(0.000)	(0.000)	(0.000)	(0.000)	(0.000)	(0.000)	(0.000)	(0.000)	(0.000)	(0.000)	(0.000)	(0.000)
Urban	-0.012	0.054***	-0.012	0.071***	-0.120***	-0.202***	0.015	0.053***	0.020	0.052*	-0.023	-0.811***
	(0.012)	(0.015)	(0.015)	(0.022)	(0.021)	(0.022)	(0.015)	(0.019)	(0.018)	(0.027)	(0.120)	(0.043)
Married	0.011	-0.018*	0.010	-0.019	0.012	-0.020*	0.004	0.000	-0.002	-0.004	0.000	-0.000
	(0.007)	(0.011)	(0.008)	(0.012)	(0.008)	(0.011)	(0.010)	(0.013)	(0.010)	(0.014)	(0.011)	(0.014)
Secondary education	0.024***	0.032***	0.028***	0.030***	0.027***	0.013	0.016	0.035***	0.012	0.036***	0.012	0.018**
	(0.008)	(0.010)	(0.009)	(0.011)	(0.008)	(0.009)	(0.012)	(0.010)	(0.012)	(0.011)	(0.012)	(0.009)
Tertiary education	0.022**	0.060***	0.029**	0.056***	0.026***	0.043***	0.056***	0.061***	0.058***	0.060***	0.057***	0.056***
	(0.011)	(0.013)	(0.011)	(0.014)	(0.009)	(0.013)	(0.017)	(0.018)	(0.019)	(0.019)	(0.018)	(0.017)
Wealth quintile 2	0.040	0.043**	0.035	0.032	0.020	0.011	0.042	0.017	0.033	0.006	0.023	0.005
	(0.027)	(0.021)	(0.029)	(0.021)	(0.032)	(0.017)	(0.028)	(0.014)	(0.034)	(0.016)	(0.035)	(0.016)
Wealth quintile 3	0.091***	0.054**	0.087***	0.048**	0.061*	0.009	0.058*	0.056***	0.050	0.040**	0.020	0.024
	(0.029)	(0.023)	(0.031)	(0.023)	(0.033)	(0.018)	(0.033)	(0.017)	(0.039)	(0.018)	(0.040)	(0.017)
Wealth quintile 4	0.091***	0.064***	0.087***	0.055**	0.054*	0.013	0.085***	0.072***	0.079**	0.055***	0.046	0.035*
	(0.027)	(0.022)	(0.029)	(0.023)	(0.032)	(0.019)	(0.031)	(0.019)	(0.036)	(0.019)	(0.038)	(0.019)
Wealth quintile 5	0.092***	0.052*	0.086***	0.040	0.053	0.006	0.073**	0.070***	0.063*	0.052**	0.028	0.032
	(0.028)	(0.027)	(0.030)	(0.028)	(0.033)	(0.025)	(0.033)	(0.025)	(0.038)	(0.026)	(0.040)	(0.024)
Tobacco user	-0.007	0.007	-0.008	0.016	-0.008	-0.002	0.022**	0.045***	0.027***	0.047***	0.027***	0.037***
	(0.007)	(0.011)	(0.007)	(0.012)	(0.007)	(0.012)	(0.009)	(0.012)	(0.009)	(0.013)	(0.009)	(0.013)
Alcohol user	-0.026***	-0.038***	-0.026***	-0.042***	-0.018**	-0.032***	-0.033***	-0.033**	-0.030***	-0.035**	-0.023**	-0.029**
	(0.009)	(0.012)	(0.009)	(0.012)	(0.009)	(0.011)	(0.010)	(0.014)	(0.010)	(0.014)	(0.010)	(0.013)
Diabetes	0.056***	0.043***	0.058***	0.048***	0.057***	0.045***	0.007	0.036***	-0.003	0.038***	-0.008	0.032***
	(0.007)	(0.008)	(0.007)	(0.008)	(0.007)	(0.008)	(0.011)	(0.011)	(0.011)	(0.012)	(0.011)	(0.012)
Depressed	-0.004	-0.020*	-0.006	-0.019	0.000	-0.011	0.034***	-0.002	0.032***	-0.004	0.039***	0.007
	(0.006)	(0.012)	(0.006)	(0.012)	(0.006)	(0.012)	(0.010)	(0.015)	(0.010)	(0.016)	(0.009)	(0.016)
Pregnant	0.080**	0.015	0.064*	0.005	0.050	0.043	0.073	0.115*	0.067	0.115*	0.038	0.102
	(0.034)	(0.061)	(0.034)	(0.056)	(0.035)	(0.046)	(0.058)	(0.066)	(0.060)	(0.067)	(0.063)	(0.073)
Recently lost job	-0.006	-0.081***	-0.008	-0.076***	-0.001	-0.072***	0.021	-0.030	0.019	-0.041	0.019	-0.041
	(0.018)	(0.028)	(0.018)	(0.029)	(0.018)	(0.027)	(0.021)	(0.025)	(0.021)	(0.028)	(0.022)	(0.032)
Obese	0.021***	0.013	0.020***	0.011	0.019***	0.013	-0.038***	-0.028***	-0.042***	-0.026**	-0.040***	-0.024**
	(0.007)	(0.009)	(0.007)	(0.010)	(0.007)	(0.009)	(0.009)	(0.010)	(0.009)	(0.011)	(0.009)	(0.010)
year5	0.031	-0.053**	0.113**	-0.096***	-0.027	0.480***	-0.007	-0.647***	0.055	-0.655***	0.088	-0.160**

	(0.034)	(0.025)	(0.047)	(0.032)	(0.063)	(0.031)	(0.035)	(0.032)	(0.045)	(0.042)	(0.089)	(0.062)
year6	-0.053*	-0.054***	-0.010	-0.084***	-0.066	0.474***	-0.122***	-0.648***	-0.068	-0.657***	-0.066	-0.137***
	(0.030)	(0.019)	(0.047)	(0.029)	(0.046)	(0.017)	(0.047)	(0.028)	(0.060)	(0.037)	(0.061)	(0.019)
year7	-0.012	-0.084***	0.027	-0.103***	-0.029	0.446***	-0.088**	-0.662***	-0.039	-0.660***	-0.046	-0.150***
	(0.030)	(0.021)	(0.046)	(0.028)	(0.044)	(0.022)	(0.044)	(0.033)	(0.057)	(0.038)	(0.057)	(0.032)
year8	-0.001	-0.096***	0.038	-0.110***	-0.014	0.483***	-0.108**	-0.548***	-0.058	-0.542***	-0.079	-0.033
	(0.030)	(0.033)	(0.046)	(0.034)	(0.044)	(0.025)	(0.043)	(0.039)	(0.057)	(0.048)	(0.055)	(0.040)
year9	-0.029	-0.131***	0.011	-0.148***	-0.028	0.428***	-0.091**	-0.619***	-0.050	-0.609***	-0.090*	-0.079
	(0.028)	(0.029)	(0.045)	(0.035)	(0.043)	(0.030)	(0.040)	(0.040)	(0.054)	(0.047)	(0.052)	(0.077)
country3	-0.108***		-0.098***				-0.105**		-0.091*			
	(0.033)		(0.038)				(0.051)		(0.055)			
country7	0.041**		0.048*				0.103***		0.096***			
	(0.018)		(0.025)				(0.026)		(0.037)			
country8	-0.088***		-0.102***				-0.011		-0.008			
	(0.025)		(0.029)				(0.029)		(0.038)			
country9	-0.052*		-0.056*				0.123***		0.118***			
	(0.029)		(0.033)				(0.035)		(0.041)			
country11	-0.006		-0.013				0.192***		0.188***			
	(0.018)		(0.025)				(0.027)		(0.038)			
country12	-0.000		0.002				0.034		0.033			
	(0.019)		(0.024)				(0.026)		(0.033)			
country13	-0.036*		-0.041				-0.051		-0.055			
	(0.019)		(0.026)				(0.038)		(0.047)			
country14	-0.109***		-0.098***				-0.000		0.007			
	(0.025)		(0.034)				(0.033)		(0.045)			
country16	-0.066***		-0.069**				0.095***		0.104**			
	(0.021)		(0.027)				(0.035)		(0.042)			
Public health facility available			-0.001	0.082***					0.058*	0.049		
			(0.027)	(0.030)					(0.033)	(0.034)		
Private health facility available			-0.010	-0.040					-0.020	0.011		
			(0.017)	(0.025)					(0.021)	(0.025)		
Any antihypertensive drug available			0.036	-0.096***					0.012	-0.076		
			(0.066)	(0.033)					(0.050)	(0.048)		
Number of antihypertensive drug available			0.004	0.007					0.003	0.010		
			(0.006)	(0.005)					(0.008)	(0.008)		
Electric street lighting present			0.041	0.039*					0.030	0.034		
			(0.038)	(0.022)					(0.033)	(0.029)		
Traffic lights present			-0.003	-0.014					0.008	-0.007		
			(0.021)	(0.021)					(0.019)	(0.030)		
Completeness of road paving			0.006	0.014					-0.014	-0.017		
			(0.021)	(0.015)					(0.023)	(0.020)		
year13	0.039	-0.063					-0.051	-0.202**				
	(0.071)	(0.173)					(0.095)	(0.095)				
year14	0.073	0.015					-0.212**	-0.458***				
	(0.074)	(0.174)					(0.092)	(0.096)				
country20	-0.032						0.232**					
	(0.076)						(0.098)					
year10		-0.188***		-0.135		-0.009		-0.619***		-0.569***		0.020

		(0.071)		(0.097)		(0.018)		(0.046)		(0.053)		(0.020)
year11		-0.183***		-0.133				-0.635***		-0.587***		
		(0.063)		(0.089)				(0.056)		(0.057)		
country2		-0.046***		-0.023				-0.133***		-0.095**		
		(0.016)		(0.026)				(0.028)		(0.043)		
country4		0.070***		0.044*				0.009		0.007		
		(0.024)		(0.025)				(0.035)		(0.037)		
country6		-0.277***		-0.254***				-0.091**		-0.065		
		(0.027)		(0.048)				(0.036)		(0.050)		
country10		0.060***		0.044*				-0.019		-0.030		
		(0.023)		(0.025)				(0.040)		(0.042)		
country15		-0.171***						-0.227***				
		(0.048)						(0.050)				
country17		0.000		0.016				-0.018		-0.004		
		(0.048)		(0.056)				(0.041)		(0.046)		
country18		-0.701***						-0.708***				
		(0.121)						(0.096)				
country21		-0.057						-0.411***				
		(0.174)						(0.096)				
year4						0.541***						0.481***
						(0.030)						(0.046)
Constant	-0.023	0.229	-0.149	0.275*	0.063	0.001	0.146	0.823***	0.038	0.860***	0.216	0.934***
	(0.151)	(0.139)	(0.192)	(0.155)	(0.174)	(0.141)	(0.199)	(0.139)	(0.217)	(0.166)	(0.240)	(0.156)
Observations	12,315	11,545	11,614	10,494	11,614	10,494	12,315	11,545	11,614	10,494	11,614	10,494
R-squared	0.071	0.071	0.072	0.068	0.041	0.020	0.051	0.054	0.050	0.054	0.019	0.009
Number of epochid					209	202					209	202

Robust standard errors in parentheses
*** p<0.01, ** p<0.05, * p<0.1
