# Supplementary material for: Does greater individual social capital improve the management of hypertension? Cross-national analysis of 61 229 individuals in 21 countries
Source: BMJ Glob Health. 2017 Dec 17;2(4):e000443. doi: 10.1136/bmjgh-2017-000443 (PMC5759715; doi:10.1136/bmjgh-2017-000443)
Supplement: Supplementary file 1 [file bmjgh-2017-000443supp001.pdf]

Hypertension management outcomes	
Detection	Self-reported to have been diagnosed by a health professional with hypertension and self-reported to be taking a specific antihypertensive medication
Treatment	Self-reported to be taking a specific antihypertensive medication
Control	Self-reported history of hypertension who had an average systolic and diastolic blood pressure of less than 140/90 mm Hg
Social capital variables	
Membership (bridging type)	Self-reported membership of any of the following: self-help group, co-operative, social club, sports club or religious group church
Trust in people (bridging type)	Self-reported strong belief that people are generally honest and want to help others, or that others will reciprocate the respect shown to them
Trust in organisations (linking type)	Self-reported strong belief that can rely a great deal on civic or religious organisations for help
Household and individual control variables	
Female	Female vs. male sex
Age	Age in years
Urban	Urban vs. rural location
Married	Currently married/common law/living with partner vs. never married/widowed/separated/divorced
Primary education	Highest level of education completed is primary or none
Secondary education	Highest level of education completed is secondary/high school/higher secondary/trade school
Tertiary education	Highest level of education completed is college/university
Wealth quintile 1	Poorest wealth quintile from asset-based wealth score
Wealth quintile 2	Middle-poor wealth quintile from asset-based wealth score
Wealth quintile 3	Middle wealth quintile from asset-based wealth score
Wealth quintile 4	Middle-rich wealth quintile from asset-based wealth score
Wealth quintile 5	Richest wealth quintile from asset-based wealth score
Tobacco user	Currently use/formerly used tobacco products vs. never used tobacco products
Alcohol user	Currently use/formerly used alcohol products vs. never used alcohol products
Diabetes	Diagnosed with diabetes
Depressed	Self-report of having felt sad, blue, or depressed for two weeks or more in a row in the past 12 months
Pregnant	Currently pregnant
Recently lost job	Self-report of having lost job during the last 12 months
Obese	Body mass index measured to be greater or equal to 30
Community control variables	
Public health facility available	At least one public hospital/clinic available within community
Private health facility available	At least one private hospital/clinic available within community
Any antihypertensive drug available	At least one antihypertensive medication (among captopril, enalapril, ramipril, metoprolol, atenolol, amlodipine, hydrochlorothiazide, furosemide) available at community retail pharmacy
Number of antihypertensive drug available	Number of antihypertensive medication categories of (among captopril, enalapril, ramipril, metoprolol, atenolol, amlodipine, hydrochlorothiazide, furosemide) available at community retail pharmacy
Electric street lighting present	Electric street lighting present in the community
Traffic lights present	Traffic lights present in the community
Completeness of road paving	No paved roads vs. partially paved road vs. completed paved roads within the community
